# Supplementary material for: The elementary forms of digital communication
Source: PLoS One. 2022 Sep 2;17(9):e0273726. doi: 10.1371/journal.pone.0273726 (PMC9439205; doi:10.1371/journal.pone.0273726)
Supplement: S1 File — (PDF) [file pone.0273726.s001.pdf]

APPENDIX A: PARTICIPANT RECRUITMENT EMAILS

EMAIL 1: SOLICITATION EMAIL

Dear X,
As you know, I am writing a senior honors thesis for my sociology concentration. My project is called the Presentation of Self in Digital Life and I am hoping to understand how college students present themselves in emails and texts. To do this, I am going to be analyzing various email and text communications from different people. I am contacting you because I am hoping you will want to participate. (I got your name from X, who I believe has talked to you about this already.) This is a really exciting topic that can help us understand how human interactions are changing for people our age and how the technologies available to us are influencing those changes. Participation in this study would involve a few separate components: you would provide me with a sample of recent emails and recent texts and we would meet once for a maximum one hour interview.

If you are interested in participating in this study, please let me know and I will give you more specific details on the things your participation will entail.

Thanks for your time,
Lauren Brown

EMAIL 2: FOLLOW-UP EMAIL

Hi X,
Thanks for getting back to me. I'm so excited that you are interested in participating. This email is a little long, so please make sure to read all of it because it contains a lot of important information.

As I mentioned in the initial email, there are three components to participating: it would require giving me digital copies of some of emails you sent over the past month, hard copies of some texts you sent, and an hour or so of your time for an interview.

Emails

1. I would like 10 emails you sent to authority figures (bosses, professors, tfs, coaches)
2. I would like 10 emails you sent to 3 close friends of the same sex (each)
3. I would like 10 emails you sent to 3 close friends of the opposite sex (each)
4. I would like 10 emails you sent to your immediate family
5. I would like 10 emails you sent to a romantic partner (boyfriend/girlfriend, someone you are dating)
   *if you do not have a girlfriend or boyfriend, you might not have emails of this nature
6. I would like 5 emails you sent to a peer who is not a close friend

Text Messages

1. I would like 10 texts you sent to authority figures (bosses, professors, tfs, coaches)
2. I would like 10 texts you sent to 3 close friends of the same sex
3. I would like 10 texts you sent to 3 close friends of the opposite sex
4. I would like 10 texts you sent to your immediate family
5. I would like 10 texts you sent to a romantic partner (boyfriend/girlfriend, someone you are dating)
6. I would like 5 texts you sent to a peer who is not a close friend

Once I have received these data, we will schedule a date for an **interview**.

Questions you may have:
What dates should these emails and texts be from?
The newer the communications, the better. If you only have communications with someone (for example, a boss) from the summer because you had an internship, even though that is not technically within the last month, because it is the most recent one you have, it is still appropriate for this research.

Do I have to give you an email or text that I am not comfortable sharing?
NO. Only provide information that you are comfortable with me reading. Remember though, that everything is completely confidential. No mention of your real name will be made in my work at any point and there will be no way for any one to associate your emails or texts with you.

What about the identity of people I'm talking to? Will they be anonymous?
Only texts and emails that *you* wrote will be used for data—in other words, if what you give me contains emails or texts written by the people with whom you were interacting, those will not be used as part of the data. The responses and identities of those people will not be used at any point in this research.

Given the information that is required of you, if you are still interested in participating, please download and read the attached fact sheet. If you have any questions, about either the research topic or methods, please do not hesitate to ask me or any of the contacts listed on that form.

And finally, if after reading this email and the fact sheet you decide you want to participate, we can set up a meeting time to conduct your interview. At this interview I will have printed copies of a consent form (that contains much of the same information as the fact sheet) with me for you to sign.

Thanks again,
Lauren Brown

P.S. If you decide to participate, I will send you a "communications checklist" so that you can make sure you're not forgetting to give me any texts or emails. And if at any point you have a question or are confused, remember that you can always contact me to clarify!

FINAL EMAIL

Hi X,
I'm so glad you have decided to participate. Thank you for your time!

Attached is a checklist for emails and texts that I would like from you. You can either give me printed copies of your emails or you can email them in a pdf/text document form. If you decide to do the latter, I will delete the document after printing it.

Also included are instructions for how you should gather your texts and emails.

As always, let me know if you have any questions and thank you again!

Thanks,
Lauren

ATTACHED CHECK LIST:

Please compile the following communications. I ask that they be from within the PAST MONTH. If this is not possible for some reason, please let me know the circumstances and we can discuss other options. Thank you!

Emails

7. I would like 10 emails you sent to authority figures (bosses, professors, tfs, coaches)
8. I would like 10 emails you sent to 3 close friends of the same sex
9. I would like 10 emails you sent to 3 close friends of the opposite sex
10. I would like 10 emails you sent to your immediate family
11. I would like 10 emails you sent to a romantic partner (boyfriend/girlfriend, someone you are dating)

    *if you do not have a girlfriend or boyfriend, you might not have emails of this nature
12. I would like 5 emails you sent to a peer who is not a close friend

Text Messages

7. I would like 10 texts you sent to authority figures (bosses, professors, tfs, coaches)
8. I would like 10 texts you sent to 3 close friends of the same sex (each)
9. I would like 10 texts you sent to 3 close friends of the opposite sex (each)
10. I would like 10 texts you sent to your immediate family
11. I would like 10 texts you sent to a romantic partner (boyfriend/girlfriend, someone you are dating)
12. I would like 5 texts you sent to a peer who is not a close friend

In order to obtain a hard copy of your text messages, call your provider and request that they send you a copy of your texts from the last month. If they ask, you can just say that you are trying to go over your text activity or and would like a hard copy, etc.

You can also go through your phone manually and write your text messages onto this sheet under each category and return it to me. Please include times of your texts and the time of the message it was replying to. For example, your returned sheet might look like this…

I would like 8 texts you sent to authority figures (bosses, professors, tfs, coaches)
5:06 Hi. I just wanted to let you know I'm running a little late. I'll be there as soon as I can.
(5:07)
5:08 I know. I am sorry. Be there soon. (Etc.)

APPENDIX B: INTERVIEW INFORMATION SHEET

PURPOSE OF THE RESEARCH:
To understand how college students present themselves in email and text communication.

WHAT YOU WILL DO IN THIS RESEARCH:
You will be interviewed about the motivations behind the substance and style of your writing. With your permission, I will record the interviews so that I can be fully engaged and not have to pause to take notes. Your name will not be recorded on the interview tape.

TIME REQUIRED: The interview will take approximately 1 hour.

RISKS:
No major risks are anticipated, but some of the questions may be of a personal nature depending on the substance of the e-mail or text. However, you can choose not to respond to any question at any time should it make you uncomfortable.

BENEFITS:
This is a way to see how the technology available to young adults like you is changing how people interact today.

CONFIDENTIALITY:
Your responses to interview questions will be kept completely confidential and at no point in the research analysis or presentation will your identity be revealed. As I will be transcribing these interviews, there is no chance of someone recognizing your voice. The recording will be destroyed once it has been transcribed and the transcript will be permanently deleted once the research is complete. You will be assigned a pseudonym that will be used in all data reporting, analysis, and discussion. There will be no way for anyone to associate your pseudonym with your identity. The list of identities and code names will be in a password-protected file on my computer that will be destroyed upon completion of my research.

PARTICIPATION AND WITHDRAWAL:
Your participation is completely voluntary and you may withdraw from the study at any time without penalty. You may also choose to skip any question during the interview if at any point you become uncomfortable.

To Contact the Researcher: If you have questions or concerns about this research, please contact: *Lauren Brown*, [phone number and email address redacted]. You may also contact the Faculty Sponsor: Jason Beckfield: [phone number and email address redacted].

For questions, concerns, suggestions, or complaints that are not being addressed by the researcher, or research-related harm, contact: *Jane Calhoun* (617) 495-5459 or jcalhoun@fas.harvard.edu Harvard University Committee on the Use of Human Subjects in Research // 1414 Mass. Ave., Room 234 // Cambridge, MA 02138.

APPENDIX C: INTERVIEW GUIDE

Background

Where are you from?
How old are you? What is your profession/what school do you attend?

Technology Use

How long have you had a cell phone? An email account?
How often do you email?
How many texts would you guess you send a day?
How often do you talk on the phone?
What is your preferred method of communication? Can you explain why? Is it recipient-dependent?
What about in the case of an argument or disagreement? What method of communication would you be most likely to use? Is there a particular reason you prefer ___? Why do you think that is?
You said that you thought text/email/phone was ___. Can you talk more about this difference?
Do you use these technologies equally (with the same frequency) with everyone? Even people you see face-to-face regularly?
If you see someone face-to-face frequently, are text and email important? Can you elaborate?
If you were making plans for next weekend, how would you do it?
What if you had to contact a professor or boss? If you had to get in touch with a family member?

Response Time

How long does it usually take you to respond to a text? An email?
What does it depend on?
I understand that these are *your* typical response times. Do you find that these are different from other people's or would you say they are universal?
So when you contact someone, what sort of expectations do have for how long it will take them to respond? Does this depend on whether it's a text or email? Or to whom the communication is sent? Or do you expect the same from everyone?
In a typical exchange, who are the people you respond to fastest? Who do you respond to slowest?
So, you say you respond to X slowest (or fastest), what do you think the reason for this is? Do you ever take a long/short (i.e. the opposite) time to respond to him/her?
In what types of situations do you respond to someone or would someone respond to you slowly?

Response formulation

Could you walk me through the process of emailing a professor—what is your thought process? What steps do you take?
What about when you're emailing a close friend—walk me through this.
It is a Saturday night and you want to meet up with a boy/girl (opposite sex). How do you go about doing this? Would you send a text or call them? What would you say? Is there a specific way you would say it?
What kind of words do you use when you text or email with your friends? Is this the same with everyone you communicate with digitally? Would you say you use "conventional" English in digital communications?

Are these the same words you would use to talk to people your age that you don't know? And what about parents, professors, etc.?

If not, what types of words would you use with them? What are your thoughts about why you don't use the same words for x vs y?

Collaboration

Do you ever use outside resources to help you write a text or email message? What types of resources—people, websites, what? Can you elaborate? How often would you say you do this?

Is it typical for people to consult ___ while writing a text or email?

Would you say it makes a difference when a friend helps you or you help a friend write a text? Can you elaborate?

If the recipient knew that the person they were emailing or texting had help, do you think they would care? Why/why not?

Do you think you would care if you found out?

What about on the opposite end—do people show each other personal messages? In what sorts of scenarios? Are there limits to this—certain people you can show, or only certain types of messages?

Would you be upset if you found out someone was showing friends a message you had sent them? Can you elaborate? What emotion would you feel?

APPENDIX D: CONSENT FORM

PURPOSE OF THE RESEARCH:
To understand how college students present themselves in email and text communication.

WHAT YOU WILL DO IN THIS RESEARCH:
You will be asked to present texts and emails you sent to different types of people.

EMAILS
- 10 emails you sent to authority figures (bosses, professors, tfs, coaches)
- 10 emails you sent to 3 close friends of the same sex
- 10 emails you sent to 3 close friends of the opposite sex
- 10 emails you sent to your immediate family
- 10 emails you sent to a romantic partner (boyfriend/girlfriend, someone you are dating)
  *if you do not have a girlfriend or boyfriend, you might not have emails of this nature
- 5 emails you sent to a peer who is not a close friend

TEXT MESSAGES
- 10 texts you sent to authority figures (bosses, professors, tfs, coaches)
- 10 texts you sent to 3 close friends of the same sex (each)
- 10 texts you sent to 3 close friends of the opposite sex (each)
- 10 texts you sent to your immediate family
- 10 texts you sent to a romantic partner (boyfriend/girlfriend, someone you are dating)
- 5 texts you sent to a peer who is not a close friend

You will also be interviewed about the motivations behind the substance and style of your writing. With your permission, I will record the interviews so that I can be fully engaged and not have to pause to take notes. Your name will not be recorded on the interview tape.

TIME REQUIRED:
Finding the emails I request should take no more than half an hour and acquiring your texts from the past month from your cellular service provider should take less than half an hour as well. The interview will take approximately 1 hour.

RISKS:
No major risks are anticipated, but some of the questions may be of a personal nature depending on the substance of the e-mail or text. However, you can choose not to respond to any question at any time should it make you uncomfortable.

BENEFITS:
This is an exciting way to see how the technology available to young adults like you is changing how people interact today.

CONFIDENTIALITY:
Your responses to interview questions will be kept completely confidential and at no point in the research analysis or presentation will your identity be revealed. As I will be transcribing these interviews, there is no chance of someone recognizing your voice. The recording will be destroyed once it has been transcribed and the

transcript will be permanently deleted once the research is complete. You will be assigned a pseudonym that will be used in all data reporting, analysis, and discussion. There will be no way for anyone to associate your pseudonym with your identity. The list of identities and code names will be in a password-protected file on my computer that will be destroyed upon grading of my thesis. I would also like to remind you that the texts and emails which you have already given to me are also going to remain completely confidential and will never be associated with any identifiers.

PARTICIPATION AND WITHDRAWAL:

Your participation is completely voluntary and you may withdraw from the study at any time without penalty. You may choose to eliminate any email or text communication from the data set if you become uncomfortable with its inclusion and you may do this, as well as withdraw, by simply informing me of your decision. You may also choose to skip any question during the interview if at any point you become uncomfortable.

To Contact the Researcher: If you have questions or concerns about this research, please contact: *Lauren Brown*, [phone number and email address redacted]. You may also contact the Faculty Sponsor: Jason Beckfield: [phone number and email address redacted].

For questions, concerns, suggestions, or complaints that are not being addressed by the researcher, or research-related harm, contact: *Jane Calhoun*, (617) 495-5459 or jcalhoun@fas.harvard.edu, Harvard University Committee on the Use of Human Subjects in Research // 1414 Mass. Ave., Rm. 234// Cambridge, MA 02138.

AGREEMENT:

The nature and purpose of this research have been sufficiently explained and I agree to participate in this study. In addition to my interview responses, I give the researcher permission to quote portions of or reprint any emails or text messages I have given her. I understand that I am free to withdraw at any time without incurring any penalty.

Participant Signature: ___________________________________

Date: _________________

Name (print): ____
