# Supplementary material for: Correction: The elementary forms of digital communication
Source: PLoS One. 2023 Oct 24;18(10):e0293575. doi: 10.1371/journal.pone.0293575 (PMC10597490; doi:10.1371/journal.pone.0293575)
Supplement: S1 File — (PDF) [file pone.0293575.s001.pdf]

PLOS ONE

RESEARCH ARTICLE

The elementary forms of digital communication

Lauren S. Brown[1], **Kevin Lewis**[2]*

1 Energy BBDO, Chicago, Illinois, United States of America, **2** Department of Sociology, University of California, San Diego, La Jolla, California, United States of America

* lewis@ucsd.edu

Check for updates

OPEN ACCESS

Citation: Brown LS, Lewis K (2022) The elementary forms of digital communication. PLoS ONE 17(9): e0273726. https://doi.org/10.1371/journal.pone.0273726

Editor: Olivier Morin, Max Planck Institute for the Science of Human History, Jena, Germany, HUNGARY

Received: March 9, 2022

Accepted: August 13, 2022

Published: September 2, 2022

Copyright: © 2022 Brown, Lewis. This is an open access article distributed under the terms of the Creative Commons Attribution License, which permits unrestricted use, distribution, and reproduction in any medium, provided the original author and source are credited.

Data Availability Statement: Due to the extraordinarily personal and sensitive nature of our data- and given that we did not anticipate pursuing publication when the data were collected in 2010, as part of the first author's senior thesis- all data (text messages, emails, and interview recordings and transcripts) were destroyed in 2011 following the submission of the thesis and in accordance with our agreement with the Harvard University IRB. Consequently, the results reported in this article constitute the entirety of the data that still exist; all other data have been destroyed. Please direct all questions to the Harvard University

Abstract

Although a tremendous amount of modern interaction is electronic, our understanding of everyday digital communications—including what they look like and how their properties vary by medium and relationship type—is still growing. In this paper, we examine digital exchange in two of its simplest forms: email and SMS. Specifically, our data consist of 2,004 messages provided by a diverse sample of college students, supplemented by in-depth interviews with their authors. These data were collected in 2010—a time when both mediums were widespread but devoid of most of their modern complexity. Based on these data, we make two contributions: First, we develop an empirically grounded typology of the basic properties of text-based digital communication; second, we document the distribution of these properties across five common relationship types. Respectively, these findings provide a starting point to understanding the substance of digital exchange in all its many forms and an empirical benchmark for comparison.

Introduction

Digital communication is now central to everyday life. 90% of American adults go online and 96% own a cellphone of some kind [1, 2]. And though digital technology is used for a staggering variety of ends—from reading news to buying products, watching videos to booking travel—for many, its core functions are social. We email colleagues, text romantic partners, and connect with friends, family, and strangers using a diverse array of social media [3].

Nevertheless, scientific understanding of everyday digital interaction has lagged behind its prevalence. Activity records contain data on the patterning of behavior on certain platforms [4–7]; surveys and interviews shed light on who uses them, how, and with whom [8–11]; and an enormous interdisciplinary literature considers how mediation impacts social exchange. Yet aside from public content, the *substance* of naturalistic digital interaction remains challenging to document: first, because findings from one platform may not generalize to others; second, because changing features and interfaces create constantly moving targets; and third, because examining private communications entails obvious practical and ethical concerns.

To help address this gap, we draw on previously unused data consisting of over 2,000 text messages and emails sent by a diverse sample of college students and supplemented by in-depth interviews with their authors—data that were collected a 12 years ago. Each of these decisions was carefully motivated. Email and short message service (SMS) messages are not

Committee on the Use of Human Subjects at cuhs@harvard.edu (617-496-2847).

Funding: The authors received no specific funding for this work.

Competing interests: The authors have declared that no competing interests exist.

only staples of everyday use, but exemplars of textual digital communication in its most basic technological and social form (i.e. alphanumeric, asynchronous, dyadic, and non-anonymous). Yet they also present variation in terms of ease (full keyboard vs. keypad), accessibility (computer vs. cell phone), and length (unlimited vs. 160 characters). College is a time when social networks may be especially diverse [12, 13] and the students in our sample were among the first generation of "digital natives," or people born into a digital world [14]. And in 2010, both email and SMS were widely used by young adults [15, 16], but still devoid of their modern-day embellishments. (For instance, our data predate the availability of emojis on standard U.S. operating systems, as well as many other features—including the ease of sending photos, videos, and animations—now integrated into smartphones.) In other words, our data feature naturalistic digital interaction, at a time when it was ubiquitous but still uncomplicated, among a sample highly comfortable with its use.

Using these data, we pursue two objectives. First, we develop a detailed, inductive typology of the properties of text-based exchange. This framework—an interpretive guidebook of sorts—provides a thorough, if necessarily inexhaustive starting point for future research interested in categorizing, organizing, and comparing digital interaction in all of its many forms. Although prior work has generated such typologies, their scope tends to be much more limited. For instance, Boneva et al. [17] describe three types of emails that sustain relationships; Thurlow [18] identifies nine functional orientations of SMS messages; and many other authors address specific communicative properties in detail (discussed below). Our aim is at once to replicate, synthesize, and extend this research.

Second, we document the distribution of these properties across five common relationship types (peers, friends, family, authority figures, and romantic partners) and the two mediums (SMS and email). Beyond their utility as interesting and (to our knowledge) novel findings in their own right, these patterns provide an empirical benchmark for a variety of possible replications and comparisons: with other populations, relationships, and communication technologies and to understand how email and text message use have changed over time. Importantly—given the vast, variegated universe comprising modern digital life and the sheer volume of studies examining its many corners—we also hope they will help illuminate what interactional dynamics may be unique to certain platforms or potentially generic to text-based exchange. In other words, we seek to exploit the *simplicity* of our data to understand modern digital communication in all of its *complexity*—a counterintuitive but familiar strategy to most sociologists [19].

This paper is organized as follows: We begin with a targeted review of relevant research, emphasizing how our approach compares to past scholarship on digital exchange. Next, we discuss our data and methods. Results are presented in two sections: one documenting the multitude of ways email and SMS are used and one demonstrating how usage varies by medium and relationship type. We then consider the larger implications of our results and conclude by returning to the modern digital context.

Context

Scholarship on computer-mediated communication (CMC) is immense and ever-growing, encompassing entire subdisciplines and scholarly journals. Here, we briefly review our overarching analytic orientation, immediately relevant prior research, and some limitations of this work.

Orientation

Three general analytic traditions are relevant to this study. First, we situate our work within the broad field of mediated intimacy. As summarized by Petersen et al. [20:2–3], mediation is

"an active process of doing and becoming, in and through media technologies." In other words, it is a reciprocal and ongoing co-constitution between humans and technology [21]. "Mediated intimacies" refer specifically to the use of digital platforms to sustain a broad range of personal ties, from platonic to romantic to familial [22–24]. It is somewhat ironic that a lens of mediated intimacy is most commonly applied to social media—platforms known for their "public displays of connection" [25]—while *private* "intimate" communication has received less attention.

Second, we draw on Walther's foundational work on the relational consequences of CMC [26, 27]. In contrast to a prior generation of "cues-filtered-out theories" [28] emphasizing the presumed richness of face-to-face (vis-à-vis text-only) exchange [29–31], Walther's social information processing theory argues that when nonverbal cues are unavailable, interactants creatively adapt to the medium—instead employing verbal cues and alternative interaction strategies to achieve comparable levels of intimacy [27, 32, 33]. In fact, the unique benefits of digital interaction (e.g. selective self-presentation; editing capabilities; reallocation of cognitive resources) may even enable "hyperpersonal" or *improved* relational outcomes relative to offline communication [26, 34, 35].

Third, given that the substance of our research consists of everyday digital micro-interactions, we turn naturally to Goffman's theory of dramaturgy [36]. Although premised on face-to-face exchange, it has been no less influential in the field of CMC; in many ways, it is *because* of its offline focus that his work provides such illuminating comparisons [37–39]. However, many applications of Goffman focus on the curation of online "exhibitions" such as personal homepages [40, 41], profiles on social media or dating websites [42–44], or "broadcasts" like posts or tweets [45, 46] as opposed to everyday digital encounters. Other research on digital self-representation takes a more institutional approach [47] or focuses on avatars in virtual environments [48]—work that is less relevant here.

Our aim is not to provide a study in applied dramaturgy or to "update" Goffman for digital life. Rather, we draw on his rich set of tools to aid our discussion of results. The following concepts are especially central: *face*, or the self-image one promotes (and works to maintain) through performances intended for a distinct audience [49]; *front*, or "expressive equipment" consisting of the setting (i.e. scenery) and the personal front (i.e. appearance and manner) [36]; *line*, or "a pattern of verbal and nonverbal acts by which [an actor] expresses his view of the situation" [49:5]; and the division of physical space into *frontstage* and *backstage* regions (respectively, where performances are given and where "illusions and impressions are openly constructed") [36:112].

SMS and email

Although analyses of textual interaction are often integral to studies of social media [50, 51], on one hand, and to broad treatments of digital communication in select contexts [52–54], on the other, we focus here on research specifically targeted at SMS and email.

Previous work has explored the patterns and motivations of teenagers' and young adults' SMS use, for whom it is the preferred means of communication [55–57]. Texting is used to maintain relationships, fostering feelings of closeness and intimacy [58]—especially through mutual disclosure [59–61]. Other scholarship examines the lexical properties of SMS [18, 62, 63], including emoticons [64] and emojis [65], and code switching and language choice among bilingual users [66, 67]. Some of this research is part of a critical debate surrounding youth and technology use—for example, showing that texting detracts from learning [68] and sleep [69] and fosters anxiety [70] and alienation [71] or instead arguing that concerns about children's texting habits are misplaced [72].

Research on email shares many of the same emphases. These include studies of how email is used to maintain ties [17, 73, 74]—especially over long distances, as with migrants [75]—and examinations of its linguistic features [76, 77] or general content and purpose [78]. Earlier work focused on email in the workplace [79]. Other notable strands of research compare email communication with phone calls or voicemails [80, 81]; examine gender differences in message content, recipients, and perceptions of usefulness [17, 82, 83]; and use patterns of email exchange to learn how social networks form and evolve [5, 84, 85].

Limitations

Although certainly not true of all studies, we offer the following broad comments on the preceding body of work. First, much prior research takes textual communications at face value without exploring their subjective meaning to authors and audiences. On the other hand, relying solely on self-report presents an equally incomplete portrait of human behavior. Comparatively few studies [38, 58, 78, 86] examine the content people produce together with their understandings of why they produced it.

Second, past research on the content of texts and emails commonly focuses on one aspect of messages, such as their "semiotic tactics" [86], degree of cultural fit [87], nonverbal cues [88], or use of relational management strategies [73] rather than exploring messages more holistically and considering substantive, stylistic, and functional dimensions in tandem.

Third, due to data access and privacy concerns—and notwithstanding a number of important exceptions [18, 58, 73, 88]—it is still difficult to examine naturalistic exchanges, i.e. the everyday messages people send and receive outside the confines of the laboratory or workplace. Fourth and relatedly, past research frequently focuses on a particular kind of interaction [89], such as among coworkers [31, 90], romantic partners [91, 92], or strangers [86] or else includes a broad set of relationships but does not systematically distinguish among them [38, 58, 62]. Respectively, these tendencies obscure the breadth and versatility of modern digital communication and ignore the basic insight that a core determinant of any performance is the audience to whom it is tailored [36, 73, 93].

As Baym [94:59] suggests, "Instead of asking what mediation *does to* communication, we can also ask what people *do with* mediated communication" (italics in original). We pursue this guidance in the remainder of this paper. Specifically, we shed light on the digital encounters of college students, using 1) a mixed-methods investigation of 2) a variety of features of 3) naturalistic text messages and emails, including 4) systematic comparisons by relationship type—identifying the distinct opportunities for connection afforded by text-based digital technology.

Materials and methods

Our methods consist of content analysis and in-depth interviews. In late 2010 and early 2011, we requested a total of 150 text messages and emails from each of 15 research subjects, yielding 2,004 distinct communications (some subjects could not provide some types of messages; see below). We coded these communications for a variety of properties and interviewed all subjects about their perceptions and usage patterns of SMS and email. Our study was approved by the Harvard University Committee on the Use of Human Subjects (where both authors were affiliated at the time) and informed written consent was obtained from all participants. Here, we describe the details and motivation of each step in our research design.

Sampling

College students are a common focus of prior research, due to both their accessibility and high rates of technology adoption. College students in 2010 also came of age during the rise of SMS

and mainstream (as opposed to merely work-related) email use and so helped establish many of the nuanced social rules surrounding these technologies. To identify potential subjects, we began by contacting acquaintances of the first author (who was then a senior in college), who in turn connected us with friends they believed would be interested in participating. Potential subjects received a series of three emails prior to participation (see S1 File). We sent the first email to 23 contacts, the second to 19, and the third to 17—two of whom dropped out—amounting to a final sample of 15 or a response rate of 65%.

Given the nature of our study, we took for granted that any type of random sampling would be impossible (insofar as very few people would be willing to share such private material with strangers *and* set aside time to be interviewed). We hoped that by targeting contacts removed by two degrees, we would harness the trust benefits of a shared acquaintance [95] while slightly reducing the biases of convenience sampling. Still, we attempted to recruit a sample that was as socio-demographically diverse as possible. Participants represented a range of racial identities (including black, white, Hispanic, and Asian) and socio-economic backgrounds (from lower-middle to upper class) and grew up in a variety of American cities and towns. They attended many different four-year colleges and had an average age of 21. Eight were male and seven were female.

Text and email collection

We requested 55 emails and 95 text messages from each participant, sent to five categories of recipients: authority figures, close friends (same- and opposite-sex), immediate family members, romantic partners, and peers who were not close friends (see overview in Table 1). These categories build on prior research [17, 73, 89, 96, 97] and represent a diversity of expected age differences, intimacy levels, and power dynamics. Securing a random sample of messages seemed as unlikely as a random sample of respondents. In our best effort to maximize data representativeness while following IRB guidelines, we clearly communicated our research goals and asked generally for "a sample" of messages. (While participants were repeatedly reassured of the confidentiality of their data, they were also clearly instructed not to provide messages that they were uncomfortable sharing; see S1 File).

Though we requested messages from the past month, in some cases older communications were unavoidable (for example, many students only interacted with a boss during the summer). Participants also reported difficulty finding 20 communications to non-friend peers, so we lowered this requirement to ten. Subjects typically copied and pasted emails into a formatted checklist we provided, but submitted text messages in a variety of ways depending on the kind of phone they used (e.g. some with iPhones uploaded conversations to their computer

Table 1. Total communications requested (received) from research participants ($N = 15$).

Interaction with. . .	Emails	Text messages
Authority figure (boss, coach, professor, etc.)	150 (150)	150 (31)
Close friend of same sex	150 (150)	450[a] (450)
Close friend of opposite sex	150 (150)	450[a](450)
Immediate family members	150 (150)	150 (132)
Romantic partner (boyfriend, "hook-up" partner, etc.)	150 (73)	150 (100)
Peer who is not close friend	75[b] (75)	75[b] (75)

[a] We asked each participant for 10 text messages to each of 3 different close friends.

[b] Because many respondents were unable to produce 10 emails or text messages to peers, we reduced our request to 5 communications of each type.

https://doi.org/10.1371/journal.pone.0273726.t001

and sent us PDFs; some with Blackberries emailed themselves conversations and forwarded excerpts; others manually transcribed their own messages). We permitted emails addressed to multiple recipients but not to a formal listserv. (Texting multiple recipients was not yet widespread.) If subjects accidentally submitted messages they had *received* as well, we immediately deleted them; we examined outgoing messages only.

Inductive coding

We followed a constructivist grounded theory approach [98] to interpret the messages through textual analysis. We let concepts emerge from the data [99] and first used open coding to develop codes inductively [100]. We then used focused coding to distill these codes in tandem with data analysis to ultimately develop four data-driven code categories: A) the intended purpose of the communication; B) the substantive and C) discursive techniques the author used; and D) the social function of the message. Respectively, these categories address the following questions: A) What is the reason for the communication, or what is the message doing? How does the author communicate that purpose, in terms of B) the information she includes and C) the way she expresses it? And D) what is the plausible impact of the communication on the relationship between its sender and recipient? To account for differences in message length and enable meaningful reporting of the distribution of codes across communications, we applied codes to entire messages as opposed to particular words or sentences. The same message could receive multiple codes within and across categories. We performed all coding using ATLAS.ti.

Interviews

The first author also conducted semi-structured, open-ended interviews with each of the 15 participants. These lasted approximately half an hour to 45 minutes and took place either in the home of the respondent or at a neutral location, like a café. Most interviews were conducted in Boston, MA, though some took place in Middlebury, VT and Chicago, IL—each of which represented the city where the respondent either attended college or grew up (and so returned for breaks). All interviews were digitally recorded and later transcribed. The interviews were intended to gauge subjects' general impressions of SMS and email exchange and understand their subjective experiences communicating through these mediated forms. Specifically, respondents were asked how frequently they used SMS and email, to whom they sent messages, and in what situations they preferred each medium. They were also asked about their perceptions of authenticity and privacy in digital exchange; their beliefs about and experiences with collaboration in message composition; their motivations for the creation of different fronts and techniques employed to do so; the impact of spatial distance from the recipient on presentation of self and teamwork; and norms surrounding digital exchange, such as meanings ascribed to response times, expectations regarding situation-dependent use of SMS versus email, and the relative formality of each. The interview guide and consent form are presented as S1 File.

The properties of text-based digital communication

We present our results in two sections. The current section documents the codes that emerged from our data, organized by the four communication properties (code families) identified above. For each code, we explain its features and provide ideal-typical illustrations. The following section of results then assesses the empirical distribution of these codes by medium and relationship type.

Table 2. Overview of communication properties and major codes.

Intended purpose
Practical information
Practical arrangement
Non-social arrangement
Corrective face-work
Non-practical information
Social arrangement
Relationship work
Substantive techniques
Qualify relationship status
Manner of address
Emotional expression
Subject matter
Discursive techniques
Abbreviations
Intentional misspelling
Extra letters
All capital letters
Emoticon or symbols
Social functions
Establish relationship
Maintain face
Maintain ties
Increase solidarity
Mutual activity
Emphasize team membership
Confirm intimacy

https://doi.org/10.1371/journal.pone.0273726.t002

For reference, a list of all major codes is presented in Table 2. Messages are copied verbatim (except for names, which have been replaced with pseudonyms throughout), so apparent typos reflect the original message. When helpful, we cite interview data supporting claims about intent. Because our aim is to generate a broadly applicable framework (rather than examine how these features are patterned, as in the next section), we present SMS and email together. Codes are introduced in each sub-section in rough order of increasing *intimacy*—a central distinction that arose inductively, as in past research [18].

Intended purpose

Email and SMS communications tend to serve one of seven purposes. Young adults use text messages and email for the most impersonal relays of information to the most private confessions, demonstrating the broad role of these communications in everyday interaction. Thurlow's study of SMS [18] produced similar categories; we identified these purposes in SMS and email alike.

Practical information. These communications deal with the exchange of pragmatic details, specific requests for information, or answers to such requests. Reading a text or email in this category, it may be difficult to discern the nature of the relationship. These messages most frequently deal with events or actions already in progress or with plans for or questions about the future, i.e. "logistics."

IP.1. I will have the first one for you by this Friday, and the next two by December 8 at the latest *(M email to authority)*

IP.2. I am charging your phone and I haven't seen your wallet yet but I'm sure it's here *(F email to romantic)*

IP.3. Should I email Caroline to let her know? *(F text to friend)*

IP.4. What shud [should] masta [master] J get? Burr [beer] and some vod [vodka]? *(F email to friend)*

IP.5. 10 rows up next to the entrance: Where are you? *(M text to friend)*

IP.6. Leaving now. Are you guys still eating? *(F email to friend)*

IP.1 and IP.2 are expressions of practical information; IP.3 and IP.4 are questions about such information; and IP.5 and IP.6 are mixtures of both.

Practical arrangement. These messages also exchange functional information. However, while practical information deals with established or projected facts, practical arrangements are not yet fixed—they require coordination between sender and recipient. They are thus "arrangements" in the traditional sense: agreement on the specifics of future plans. They ask or answer the questions "Where?" (IP.7), "When?" (IP.8), or both (IP.9).

IP.7. Let's do starbucks yea!!!! *(F text to friend)*

IP.8. so i should get there 5:45ish yea? *(M email to family)*

IP.9. I think lunch at 12 is just fine time wise. Sound Bites sounds great. *(M email to authority)*

Non-social arrangement. This category (along with the more intimate "social arrangement," below) also refers to arrangements but surpasses logistics. This includes the initial expression of a desire to meet, discussion regarding the purpose of a gathering, and any *non*-recreational plan-making; arrangements in this category serve a functional purpose in the lives of their senders.

IP.10. I wanted to talk to you about potentially interviewing you for my thesis. *(F email to authority)*

IP.11. Do you think we could set up a time to meet briefly next week to go over what I will have missed? *(M email to authority)*

Both IP.10 and IP.11 are about plans for formal meetings. In many instances (e.g. IP.11), this purpose is expressed in conjunction with a practical arrangement. This may occur between individuals who do not interact frequently and thus engage in economical exchange, meaning they include multiple elements of plan-making to eliminate unnecessary dialogue.

Corrective face-work. These communications attempt to repair a lapse in performance on the part of the sender—either by conveying explicit remorse (IP.14) and/or attempting to absolve the sender of any accountability for a past, present, or predicted behavior (IP.12, IP.13).

IP.12. sorry didnt respond earlier absolute crazay week here *(F text to peer)*

IP.13. I really didn't want to just email you like this. . . I have tried to stop by your office several times this week, but you haven't been there. *(F email to authority)*

IP.14. Hey, so sorry to not have texted earlier *(M text to friend)*

Corrective face-work can occur on its own or with other purposes. When recoveries were embedded in messages with other purposes, closer inspection often suggested that these purposes were superficial vehicles for the deeper objective of corrective face-work.

IP.15. Hi Professor Cohen,

I hope that the week has been treating you well! *I am so sorry for not stopping by today as I had said I would. I ended up shopping a seminar last minute that just let out* [italics added]. I will be taking the WGS class that I was telling you about, as well as an English class this semester.

I am off to meet Sam now—once again thanks so much for putting me in touch with her!

Talk to you soon,

Stephanie *(F email to authority)*

IP.15, for example, relays non-practical information (see below) by keeping the participant's professor updated on her plans for the semester. However, recovery was the driving motivation behind this communication; Stephanie included the other details to obfuscate her aim of corrective face work "because otherwise it would seem like I was just writing to cover my ass" (Interview 6).

Non-practical information. Communications in this category convey inessential facts or details. These range from gossip to jokes to personal details irrelevant to the recipient (like Stephanie's class selection, above). These messages do not have a practical objective and typically contain jovial, sociable qualities indicating the exchange of information for the mere pleasure of exchange.

IP.16. nah bro. sshit was delicious *(M text to friend)*

IP.17. So the Woman of the Year parade was yesterday, and there are some

HILARIOUS pictures on boston.com <http://www.boston.com/ae/celebrity/gallery/hastypudding2010?pg=5> [picture included] *(F email to family)*

Social arrangement. Social arrangement messages discuss recreational plans, such as getting meals, going out, or what to do over the weekend. Such communications include a wide range of intentions and levels of specificity. They could convey a general desire to have plans in the future:

IP.18. Yay! I'm so glad they liked it. I loove Cragie on Main [a local restaurant]. Speaking of which, we should have dinner soon. I have been sick/insanely busy lately, but should be able to make time soon. When would be good for you? *(F email to friend)*

Or ask about availability for specific, unconfirmed plans:

IP.19. yooo

my moms is in town this weekend. would you be free for an early dinner on saturday? lemme know. *(M email to friend)*

Or describe thoughts regarding already established plans:

IP.20. still down for sat? you're my only plans;) lets maybs [maybe] have a chill smoking sesh [session]? *(F text to friend)*

Or exchange information about current activities:

IP.21. Hello dudettes,

I am at Kong [local bar]. One of the only girls but it's getting better. Any interest in coming? Feel like I can't leave but want to make it more interesting. *(F email to friends)*

Relationship work. These messages help maintain existing relationships—showing interest in the location, activities, and wellbeing of their recipients.

IP.22. Omg omg [Oh my god oh my god] please come home! My bday was a blast, totally understated and classy. Except for me vomiting uncontrollably for 10 minutes. My sibs came out for the weekend and that was fun as hell. We. . . drank expensive wine [text omitted]. Really ridic [ridiculous]. I'm about to go to the gym and read the new People about that girl that was kidnapped for 18 years. So excited. Love you!!!! *(F email to friend)*

IP.23. hows your paper going? *(M text to friend)*

IP.24. Hi dad,

Haven't spoken in a few days and thinking of you. How are things going? Are you excited for Sarasota? Want to speak tonight around 9 30 or ten?

Love you

Stephanie

P.S. No, nothing is wrong, and no, I'm not asking you for anything. Genuinely just saying hi *(F email to family)*

IP.25. Oh nice! Got one tomorrow too, at 2pm. Good luck madre, I'm sure you'll crush it." *(M text to family)*

IP.26. oh my god!!! im about to go to my last day of classes. And my dad is coming this afternoon. Cant wait.

I got your postcard. It made me the happiest ever.

Hows America?

Love you miss you.

MM *(M email to romantic)*

IP.27. Dammnnn 7 pg? Impressive. Keep goin, ill have alc [alcohol]/sex waiting for you wen you reach 11. Kkkkk?? *(M text to romantic)*

Whether sent to a friend (IP.22, IP.23), family member (IP.24, IP.25), or romantic partner (IP.26, IP.27), participants expressed enough intimate knowledge of the daily goings-on of their interactants to show unprompted sympathy, affection, and genuine curiosity about their lives.

Substantive techniques

While some scholars have argued that basic textual communication lacks social and emotional cues, our data revealed quite the contrary. In emails and text messages, implicit information is paralinguistic, rather than non-verbal (as in face-to-face interaction). In the absence of facial and tonal cues, body language, and physical props, individuals compensate with purposeful choices in writing style, composition, and expression [27]. This sub-section deals with four substantive techniques regarding *what* kind of information is communicated. The next, on discursive techniques, discusses *how*.

Qualify relationship status. Codes in this category situate the relationship between sender and recipient. While not all communications—particularly those that were part of an ongoing exchange—contained such statements, those that did took one of three forms:

First, in messages establishing contact with a previously unknown person, participants engaged in an *introduction*. Unlike some polite face-to-face introductions that occur when individuals happen to find themselves together, text and email introductions are motivated by specific functional purposes such as a job hunt or class enrollment. (Otherwise, the message wouldn't have been sent).

ST.28. My name is Penelope Miller and I am a junior undergraduate studying computer science at Harvard University. *(F email to authority)*

Second, when communicants already know one another but are not in frequent contact, a *reference to a past encounter*, either digital or face-to-face, may be used.

ST.29. To put a face to a name, we briefly met during a book signing at Harvard in October, when you came to speak about Half the Sky [book title] (although considering the number of people who were there, I'm not sure that this will trigger much of anything!) *(F email to authority)*

Third, participants made *reference to a future encounter*. Such messages contain explicit expressions of hope for continuing the relationship and/or interest in the recipient's response.

ST.3o. Looking forward to hearing back from you. *(M email to authority)*

ST.31. Can't WAIT to see you on Friday! *(M text to friend)*

Each of these techniques situates the communication for the recipient in terms of a (nascent, ongoing, or projected) narrative with its sender.

Manner of address. These codes refer to how subjects addressed recipients. Often, there was no address at all; but if there was, its nature helped establish the tone of the message.

If a participant addressed a recipient by title (e.g. Mr., Professor, Dr.), we considered it a *formal address*. Subjects used "Hi," "Hello," and "Dear" interchangeably and, on occasion, excluded a greeting and merely began with the recipient's name. In all such cases, the communication structure was formal as well, including a traditional sign off such as "Best," "Sincerely," or "Thank you."

ST.32. Dear Professor Rosen,

. . .

Best,

Christine *(F email to authority)*

Unlike formal addresses, *nicknames or terms of endearment* were often present throughout messages, acting as tokens of intimacy rather than respectful salutations [58]. Although nicknames (ST.33, ST.34) are individualized while terms of endearment (ST.35, ST.36) may be generic, we group them together given their functional equivalence. In some relationships, otherwise insulting names take on positive meaning (ST.37); in the privacy of light-spirited digital rapport, they are markers of closeness and affection (by highlighting the fact that the relationship is close enough to permit such words in jest).

ST.33. Neensypoo where ah youu *(F text to friend)*

ST.34. will let teddmeister know *(M text to friend)*

ST.35. Hey puffin bear. . . *(M email to romantic)*

ST.36. hi lovely!. . . *(F text to friend)*

ST.37. hussy! I miss you slut *(F text to friend)*

In sum, distinct forms of address convey the sender's perception *of* their relationship with the recipient *to* the recipient: one establishing social distance and one fostering closeness. One participant categorized all digital interactions based solely on this distinction, asserting "There's two kinds of things: dear so and so and then you sign your name. And then there are the ones that are one line or whatever without the formality" (Interview 6).

Emotional expression. Without tonal cues, facial expressions, and body language, text-based digital interactions rely heavily on the explicit verbal expression of information taken for granted in person. Subjects expressed this information through purposive diction and overt articulation of feelings [33, 101]. These tools are among the most important for digital interactants because, more than any other substantive technique, they influence how cointeractants interpret their words.

Because we identified so many distinct emotional expressions—as with subject matter, below—we summarize them in Table 3. This category encompassed the following 12 codes: deference; personal detail; gratitude; relatability; well wishes; enthusiasm; empathy; check-in; support; for you. . .; "love"; and romantic expression.

Subject matter. Unlike emotional expressions, which involve explicitly voicing feelings, subject matter refers to the focal content of a message—not unlike "what people talk about" offline [102]. This category encompassed the following 11 codes, summarized in Table 4: confirmation or agreement; apology; excuse; humor; banter; micro-coordination; link; gossip; collusion and secrets; sexual matter; and derision.

Discursive techniques

As scholars have long noted, the lack of sound or graphical content even in exclusively text-based CMC has not prevented users from finding other creative ways to increase its richness and illocutionary force [27, 58, 103]. Discursive techniques are paralinguistic elements of textual communications used to modify meaning and convey emotion [101, 104]. Traditionally

Table 3. Types of emotional expression.

Code	Explanation	Examples
Deference	Encompasses a variety of expressions (e.g. respect, a relaxed personality, indifference, ambivalence); enables the recipient to feel comfortable and make decisions favorable to her own interests. It is also, consequently, a face-saving mechanism: By deferring to the recipient, the sender ensures she will not make the wrong choice.	ST.38. Should I come to your office or should we meet elsewhere? Whatever you prefer! *(M email to authority)*<br>ST.39. i was thinking a big dinner, we could either stick to the square and do a takemura [local restaurant], border [local restaurant] or some other thing orrrr we could venture out of the sqaure [school location] and maybe do fajitas and ritas [local restaurant] or find another cool restaurant its up to you! *(F email to friend)*
Personal detail	Though not technically an emotion, including a personal detail about the recipient reflects thoughtfulness and familiarity. Refers specifically to personal details in otherwise impersonal messages; otherwise, they are less deliberate inclusions than standard aspects of intimate exchange.	ST.40. I hope this email finds you well and enjoying your winter vacation with your family (whether still in the States or already in Germany). *(M email to authority)*
Gratitude	Without facial expressions or physical reactions, appreciation is much harder to discern unless explicitly expressed. Includes recognition of already completed (ST.41) and tentative or unconfirmed (ST.42) actions.	ST.41. Thank you for making me buy this sweater!! *(M text to friend)*<br>ST.42. I would be grateful to hear from you about your willingness to take me as your student! *(F email to authority)*
Relatability	Emphasizing a shared quality or interest helps ease potential tension and establish a tone of familiarity and closeness. Takes many forms, ranging from stressing personal details senders share with their recipients (ST.45)—like interest in the same research area (ST.44)—to general expressions of shared experience like enjoying a long weekend (ST.43).	ST.43. Hope you've been enjoying the weekend (finally some lovely weather)! *(F email to authority)*<br>ST.44. I am particularly interested in learning more about your work with the Na+/Ca2+ exchanger *(F email to authority)*<br>ST.45. Get some SLEEP (this is actually the latest I've ever been up on a weeknight at Harvard- no joke- I'm a huge luzr [loser]) *(F email to peer)*
Well wishes	Articulations conveying hopes of happiness for the recipient. Typically general expressions that could be voiced to anyone and refer to the future (ST.46), but sometimes expressed regarding a past event, hoping it went favorably. Occasionally combined with a personal detail to express a personalized message of good fortune (ST.47).	ST.46. HAPPY THANKSGIVING! *(M text to friend)*<br>ST.47. Happy shopping!! *(F email to family)*
Enthusiasm	Behaviors like smiling, eye widening, and extreme inflection are so fundamental to conveying authenticity that their absence can potentially undermine a digital performance; this is overcome by obvious expressions of eagerness and passion. (They also encourage a response by making the recipient feel important or enthusiastic about a topic herself.)	ST.48. It was so exciting to see what all my physics friends will be working on. Ive been away from physics for a while but I am really excited for my thesis work. *(M email to authority)*<br>ST.49. OMG YES YES YES YES OMG I LOVE YOU thanks so much for inviting me!!!!!! *(F text to friend)*
Empathy	Demonstrations of understanding and appreciation for the feelings or circumstances of the recipient (but, unlike relatability, without associating them with the sender's own)—tactfully making the sender seem more compassionate, insightful, and sensitive.	ST.50. I hate to ask because I know you are very busy. *(F email to authority)*
Check-in	Solicitations of information on the recipient's status or wellbeing. While some are specific, such as asking how studying is going, many are general or vague; their very fact of being sent signifies closeness and concern.	ST.51. what's up *(M text to friend)*<br>ST.52. Good morning!!! How did it go? Did you love it? Hate it? Are you exhausted? Drenched? I hope you loved it. Call me! *(F text to romantic)*
Support	Messages sent to encourage recipients, show the sender is thinking of them, and express love. Unlike well wishes, they are specific to the recipient—relying on deep knowledge of her life and current stresses or obstacles in it.	ST.53. Yayayayayayayayayayaya! Proud of you. *(F email to family)*<br>ST.54. Don't be! You had the opposite of a lazy day the past three days, so you deserve it. *(M text to romantic)*
For you. . .	Instances when subjects send a piece of information—whether an anecdote, web link, or unprompted favor—they think the recipient will enjoy. Sometimes senders directly state that they are "thinking of you," but usually this is implicitly evidenced by how thoughtful and personal is the "gift."	ST.55. Hi mom! Did some searching on Rent the Runway [shopping website] and found a dress you might like? It has sleeves and is flowy and is very simple. . . The "Carrie Dress" is the one I thought you may like. *(F email to family)*
"Love"	Messages in which senders tell recipients they love them—whether that love is romantic, friendly, or familial and expressed directly (ST.56) or attached almost as an afterthought (ST.57)—less asserting than confirming affection.	ST.56. I love you! Croo! *(F text to romantic)*<br>ST.57. Let's talk soon. Much love xx *(M text to friend)*
Romantic expression	Passionate messages between romantic partners, whether innocent (ST.58) or explicit and sexual (ST.59). Because subjects are not faced with the same threat of embarrassment or rejection of a face-to-face romantic interaction, they are more fearless about expressing sexual or erotic emotion.	ST.58. I love you. Colgate is hard without you. *(F email to romantic)*<br>ST.59. Nice pic. Except now im hard [erect] at work cant stop thinkin bout your sexy bod. *(M text to romantic)*

https://doi.org/10.1371/journal.pone.0273726.t003

Table 4. Types of subject matter.

Code	Explanation	Examples
Confirmation or agreement	Messages that confirm (ST.60) or agree with (ST.61) previous statements made by the cointeractant, whether this is the whole point of the message or expressed as part of a larger communication.	ST.60. Yuuup the one in the garage *(M text to friend)*<br>ST.61. That works for me! *(F email to friend)*
Apology	Acknowledgments of offense or failure, exchanged when engaging in corrective face-work. Most directly reference the committed offense, as if to remind the recipient of why the sender should be regretful.[a]	ST.62. Oh crap sorry. I thought you had your talk today. My b [bad]. *(F text to friend)*
Excuse	Also particular to corrective face-work. Senders use the skeleton of a supposed apology to absolve themselves of responsibility—attributing the cause of the wrongdoing to forces beyond their control[a]	ST.63. Unfortunately, I won't be able to make it to class today due to a very bad cold, which has me stuck in bed. *(F email to authority)*
Humor	Jokes, sarcasm, and repartee. Regardless of their purpose, electronic messages frequently have a lighthearted, jovial tone to offset their potential impersonality; humor creates an impression of closeness and familiarity.	ST.64. Nicoles eating blow pops again. Tell her it's not medicine if it's sold at hidden sweets. *(F text to friend)*<br>ST.65. Although I have no idea who the guy we are talking to in the second picture is. . . I hope he wasn't the object of our "find the attractive people" game *(F email to friend)*
Banter	Typically part of a continuous conversation characterized by a rapid exchange of friendly remarks. Akin to inconsequential small talk—though not necessarily as polite—these messages do not relay essential information, but appear to be conversation for conversation's sake and resemble face-to-face communication much more than writing.[b]	ST.66. 11:36 am Hahah what happened? She come back? [11:38 am response] 11:39 am Haha nooooo. Devastating. [11:39 am response] 11:39am Gotta get her over for a weeknight dinner and scented candles affair. [11:40 am response] 11:40 am Hahah you can salvage that easily. She's a freshman and you're 35. *(M text to friend)*
Micro-coordination	Discussions of miniscule details about arrangements in the immediate future, including changing plans already in progress (ST.67), confirming precise times or locations (ST.68), notifying a recipient you will be late (ST.69), or employing one technology to plan the use of another.	ST.67. yoyo I'm leaving now I'll be at subway in 10. *(F text to romantic)*<br>ST.68. im inside to the left *(M text to family)*<br>ST.69. kk, kool. sorry, yea. gonna be just a lil [little] late! im leavin in like 10 or less i prom [promise] *(F text to friend)*
Link	Here, links to websites and articles either comprise the entire body of the message (ST.70) or there is also a short contextualization (ST.71), typically citing a reason for sending it. Links come in two forms: those sent without the expectation of a response, like a humorous video, and those accompanied by a question eliciting feedback, like a link to a hotel website with the question, "How does this look?"[c] A notable trend among women, in particular, is to seek feedback on a potential purchase (ST.72). Links frequently overlap with "for you" emotional expression.	ST.70. http://www.theonion.com/articles/ south-african-vuvuzela-philharmonic-angered-by-soc,17625/ *(M email to family)*<br>ST.71. Subject: Must Watch from my little cousin's fbook account- he's a 7th grader at columbia. . . http://www.youtube.com/watch [URL suppressed] *(M email to friends)*<br>ST.72. Subject: two questions<br>1) do we like these shoes? http://www.bergdorfgoodman.com/ store/ catalog/prod [URL suppressed] 2) do i have any business wearing them? (my main concern is how high the heel is) *(F email to friend)*
Gossip	Inessential information about individuals who are not involved in the exchange. In some cases, interactants witness the reported incident and in others, they hear about it secondhand. Like face-to-face gossip, interactants sometimes mock or ridicule the person(s) being discussed.	ST.73. Subject: Re: Question<br>Haha yes. She was actually at your thing on Friday. Don't know her personally, but know of her. She lives in [undergraduate residence], very off the map, sings in an a capella group, and has a long-time boyfriend (who also lives in [residence]) last I checked. Think she's incredibly good-looking though. *(M email to friend)*<br>ST.74. 11:07 am She flies from stanford to mit every like two weeks [11:07 am response] 11:08 am Pumped abt [about] having a boy? Love? Idfk [I don't fucking know] [11:09 am response] 11:11 am idk [I don't know] it was weird *(F text to friend)*
Collusion and secrets	When interactants surreptitiously convey information they do not want others to know; digital communication is thus used to both confide in cointeractants and ensure privacy in these exchanges. We group collusion and secrets together because information frequently straddles the line between disclosure of confidential information (secrets) and putting that information to use for an intended clandestine purpose (collusion).	ST.75. Hey Mom—any ideas what Ron would like? Can you give him a call, but don't want to make it too obvious. Let me know if you have any potentials. *(M email to family)*<br>ST.76. I like this idea. I'm going to speak with margaret (don't worry, I'll be subtle). How much money would need to be fundraised out of curiosity? *(F text to friend)*
Sexual matter	Some messages contain sexual innuendos, which are not obviously erotic but include suggestive undertones frequently accomplished by wordplay. Others are explicitly sexual. This substance is not necessarily flirtatious or instrumental towards a sexual goal, however; sex is a frequent subject matter aside from such "romantic work."	ST.77. And then we can awkwardly hook up sometimes when we're drunk. That's always fun. *(M text to romantic)*<br>ST.78. I need to get laid. *(F text to friend)*<br>ST.79. damn. . . i fucked up this whole picture thing. I am by farrr the worst aspiring porn star in the world. *(F text to friend)*

(*Continued*)

Table 4. (Continued)

Code	Explanation	Examples
Derision	Negative remarks about one of the interactants or about a third party; sometimes present alongside gossip, but also expressed independently. If about an interactant, messages are either self-deprecating for the sake of humor (ST.80) or written out of teasing friendliness (ST.81).	ST.80. Umm duh I know who they are. Every bleeding heart recycling lesbian tranny [transexual] liberal does. *(F email to friend)* ST.81. She looks burned in that picture. The word that comes to mind is menopausal. *(M text to friend)*

[a] Many participants conveyed a preference for expressing both apologies and excuses over SMS or email, rather than face-to-face—a finding we return to in the discussion.

[b] Indeed, many subjects felt that digital exchanges could be just as much of a conversation as face-to-face exchanges. One participant said, "I don't see the difference—you're going back and forth the same and you're just talking in written form" (Interview 11). Participants overwhelmingly perceived continuous, rapid-fire exchanges like ST.66 as "basically the same thing" as in-person encounters, which accounts for their apparent triviality and geniality.

[c] One participant articulated the distinction between links that do and do not require responses. He explained, "If I sent an email to my sister of a stupid YouTube video. . . I know that she'll laugh when she reads it, I don't need to hear that via email" (Interview 8). The purpose of these messages is typically to entertain the recipient and link-senders can feel they have accomplished this without explicit confirmation—presumably because they feel sufficiently confident of the recipient's tastes.

https://doi.org/10.1371/journal.pone.0273726.t004

associated with personal style, they can also indicate a purposeful change in register or tone. Some, however, are borne of mere convenience [18]. Discursive techniques almost always represent informality; exemplary of this is one participant's confession that if anyone besides a friend or close family member saw her typical digital writing style, "they would think I'm not good at English" (Interview 7). We identified five such techniques in our data.

Abbreviations. Abbreviations are common in text messages and emails—as on the internet at large [105]. They convey a sense of casualness, depend on the subject's writing style rather than an intention to alter meaning, may be specific to a particular in-group, and result most likely out of ease [18, 58]. They include contractions ("wk" for "week"), acronyms ("omg" for "oh my god") and shortenings ("bro" for "brother").

DT.82. see you this wknd [weekend]? (M *email to family)*

DT.83. be back in chi [chicago] dec [december] 12 *(M email to family)*

DT.84. perf [perfect] just got here *(F text to friend)*

Subjects emphasized the informality of abbreviations, as in the following quotation: "If you're in an academic setting, you don't want them to think you're dumb, so obviously you proofread and use real words like a grown up" (Interview 2). They are interpreted as a sign of "speed" (Interview 13) and indicate the message is "unofficial" (Interview 6).

Intentional misspelling. Participants use misspellings [58] to convey personal registers and make the tone of a message more representative of their own speaking. The presence of such choices is user-dependent and, unlike other discursive techniques, they are more a tool for personalization than for emphasizing certain details or expressing specific emotions. Examples include eliminating letters ("hav" for "have"), atypical spellings ("shud" for "should"), and accent representation ("sumfin" for "something").

DT.85. lez [let's] do sumfin [something]! *(M email to friend)*

DT.86. spanks spanks [thanks thanks]! *(F text to friend)*

Extra letters. Some messages contain words with extra letters that stretch out traditional spellings. This technique is used to emphasize specific words or sounds or the authenticity of a

communication's expressed emotions and also to convey friendliness, informality, and intimacy [58, 106, 107].

DT.87. Saaaaaaddnesssss *(M text to friend)*

DT.88. hayyyyyy pretttttyy lady! *(F email to friend)*

All capital letters. Typically, this technique is applied to specific words, but on occasion subjects wrote entire messages in capital letters—creating emphasis or expressing enthusiasm [58, 107].

DT.89. Don't tell mom EVER. *(M email to family)*

DT.90. Yayayayayayayay OMG OMG [oh my god oh my god] I LOVE YOUUUU *(F text to friend)*

Emoticons or symbols. Emoticons are facial representations created by sequences of punctuation, such as ":)" (a smile), and symbols are sequences of letters and numbers that represent a gesture or emotion, such as "xoxo" (hugs and kisses). A precursor to emojis, they are generally employed to communicate, clarify, or emphasize sentiment, often by mimicking physical expressions or objects [103, 108–111].

DT.91. Ok when im done with this chap [chapter] maybs [maybe]. I think im going to have to work tonight: ([sad face] *(M text to romantic)*

DT.92. love you miss you <3 [heart] *(F email to friend)*

Social functions

This category of codes addresses the micro-consequences of digital communications, or the impact of messages on the relationship between the people exchanging them. Here, elements beyond the overt contents of communications are as important to their classification as the more obvious features presented above. Though we did not solicit data on hostile relationships (e.g. "enemies"), all messages provided by our respondents appeared to augment, rather than weaken, social connections. Because these functions are accomplished by a wide array of communication types—and to underscore this point—we exclusively reference earlier examples and introduce no new data at this stage.

Establish relationship. Digital messages frequently serve as inaugural communications between unfamiliar parties. Because of the privacy and flexible time horizon in which they can be crafted, they are often a more comfortable way to begin contact compared to the pressure of real-time introductions. ST.28 illustrates such an introductory email with a potential employer, whom the participant said she would have felt too "awkward" and "nervous" to approach in person or on the phone (Interview 2). Importantly, email thus eliminates the necessity of a physical meeting—which can be inconvenient or impossible—to begin a relationship. For instance, the participant in ST.42 is inquiring about potential opportunities for graduate school. She commented that it would have been much more difficult to contact professors at various institutions had email not been an option (Interview 12).

Maintain face. A common function of messages is to help senders preserve their self-image [58]. However, this is not limited to messages whose *purpose* is corrective face-work (e.g. IP.12-15, ST.62-63). In all digital interactions, participants make discursive and

substantive choices in keeping with their role. Consequently, many messages not only serve their intended purpose, but also play a strong role in face maintenance. For example, IP.4, which employs a range of discursive techniques and focuses on a social subject—in this case, buying alcohol for a gathering—preserves the subject's already established "friend front," i.e. one that is lively and social. Conversely, messages like ST.38, ST.48, and ST.50 preserve a different kind of image by revealing their sender to be respectful.

Maintain ties. For people who cannot see each other frequently, emails and text messages are an important part of staying in touch. In ST.24, for example, the participant reaches out to her father, maintaining a close connection despite being physically distant. Digital communications also allow subjects to acknowledge important people in their lives without a large time commitment. For example, the playful tone and simple acknowledgment "I miss you" in ST.37 convey that the sender thinks about the recipient and wishes she were nearby. Even the simplest message—a link, for example (IP.17, ST.70), or a quick well wish (ST.46)—indicates to the recipient that the relationship is important. Communications like this can be sent to anyone, at virtually any time, making ties that might otherwise be forgotten or neglected (e.g. a grade school friend or a great aunt) easy to sustain.

Increase solidarity. Digital communication increases solidarity in multiple ways. On one hand, it facilitates high rates of interpersonal contact, enhancing familiarity and cohesion. On the other hand, it allows individuals to discover and emphasize similarities with each other that may not be noticed or acknowledged in person. For example, in ST.44, the participant expresses to her professor interest in the Na^+/Ca^{2+} exchanger. She goes on to explain this interest in detail—something she does not have the opportunity to do during regular in-class interactions (Interview 1). Banter, gossip, derision, collusion and secrets, and expressions of confirmation or agreement are other common means by which individuals feel close and affirm like-mindedness—occasionally by exclusion (see below).

Enable mutual activity. Text messages, and particularly emails because of their unlimited length, allow individuals who are not physically copresent to engage in mutual activity; beyond passively reporting on what one is doing or experiencing [89], others can actually be *involved*. In ST.39, for instance, the cointeractants are planning a party. Another example is collaboration in online shopping: By sending a link to a shopping website, individuals can seek the advice of friends before making a purchase (ST.72). Indeed, digital technology facilitates such exchanges even when they are unnecessary; of emails like ST.72, one subject said, "It's an excuse to talk to someone if you didn't have other particular things to say" (Interview 12). While mutual activity clearly contributes to solidarity, it also creates the special feelings of unity and accomplishment that accompany task completion.

Emphasize team membership. The basic act of digitally communicating with multiple recipients serves an important function for teams. Group messages go above and beyond enhancing solidarity by explicitly distinguishing insiders from outsiders—boundaries unlikely to be so demarcated in person. By sending group messages, the inner circle acknowledges to themselves their own insider status. Furthermore, such messages create a safe, "virtual backstage area" where team members can speak freely and act without concern for any audience. For example, both ST.71 and IP.21 were sent to multiple recipients. In ST.71, the author mocks his cousin; in IP.21, the author confesses she is not having a good time at the bar but feels a social obligation to stay. Beyond group messages, gossip and derision also mark team boundaries in less obvious ways; by alienating those being discussed, interactants bring themselves closer together and confirm membership to the same 'side.' ST.71 is an example of this, as are ST.74 and ST.81.

Confirm intimacy. Finally, individuals use digital interaction to affirm the closeness of relationships and express affection. In ST.55, this expression is implicit: The sender goes out of

her way to find dresses she thinks the recipient would like. In ST.58 and ST.59, it is explicit: The sender openly professes feelings of love or sexual attraction. The ease and accessibility of SMS and email are again important because they allow users to quickly send short messages of love, even in times apart (as in ST.56). That these messages are written proves especially crucial. A typical example of this is ST.57; the sender ends a text message coordinating a plan to "talk soon" by including "Much love xx." By compensating for the lack of corporal and tonal signifiers (if these friends had been in person, perhaps they would have simply hugged goodbye), individuals may end up explicitly highlighting emotions that would otherwise go unmentioned—thereby heightening affirmations of intimacy [26].

The distribution of properties by relationship type and medium

In the previous section, we drew upon respondents' SMS and email communications and explanations thereof to develop a set of tools to distinguish, classify, and analyze their exchange. Employing these tools, we now examine the characteristic features of communications across five types of relationships and both mediums. We begin each sub-section with a summary description; present detailed statistics on the prevalence of specific codes; and conclude with an exemplary email and text message of relationships of that type to provide a holistic, qualitative illustration of the patterns we have described. The complete distribution of codes by medium and relationship type is presented in Table 5.

Authority figures

The most common authority figures with whom respondents communicated were professors. Messages to authority figures are characterized by formality, exchanging practical information, and maintaining relationships, particularly manifested in corrective face-work. They primarily serve to heighten solidarity and maintain face. Because the informality of text messages is incompatible with the front that students are here trying to maintain, almost all data in this sub-section come from emails.

Intended purpose. Of the 181 total communications with authority figures (150 emails and 31 texts), the most frequent purposes were to exchange practical information (44% of emails, 74.2% of texts), engage in corrective face-work (28.7%, 35.5%), and make a practical arrangement (20.7%, 9.7%). Many face-work emails refer to mistakes made in person, such as:

"I'm sorry for not stopping by today, as I had said I would." (Excerpt from IP.15)

Email is a recovery tool with small costs: It takes little time and energy to craft a message that could recover face. Indeed, one wonders whether the student may have been more likely to visit the professor's office if apologizing for *not* doing so was not so easy.

Only four participants sent texts to authority figures. In all instances, the recipients were subjects' bosses at on-campus jobs and young adults themselves—suggesting a potentially important interaction between relationship type, age differences, and digital medium.

Substantive techniques. Less than half (42%) of emails to authority figures qualify the relationship. The majority of these reference a future (28%) or past (8.7%) interaction, and very few (5.3%) make introductions. This indicates subjects tended to contact authority figures with whom they already had a relationship—though not necessarily a close one. In fact, in 141 of these emails (94%), subjects addressed the recipient formally. 120 (80%) express gratitude and 43 (28.7%) express deference. Other common emotional expressions are well wishes (27.3%) and relatability (18%), reflecting a desire to strengthen the social tie. The most

Table 5. The distribution of codes by medium and relationship type.

	Authority figures		Peers		Family		Friends		Romantic partners	
	Email	SMS	Email	SMS	Email	SMS	Email	SMS	Email	SMS
Intended purpose										
Practical information	44.0	74.2	66.7	32.0	40.7	32.6	22.3	9.3	9.6	13.0
Practical arrangement	20.7	9.7	9.3	10.7	6.0	9.1	8.3	3.8	4.1	3.0
Non-social arrangement	16.0		1.3	6.7			0.7	0.1		
Corrective face-work	28.7	35.5	26.7	4.0	5.3	9.1	8.0	6.4	4.1	3.0
Non-practical information	4.0		2.7	38.7	32.7	36.4	21.3	16.9	35.6	17.0
Social arrangement		6.5	8.0	6.7	8.7	5.3	11.3	10.2	11.0	12.0
Relationship work		3.2	4.0	6.7	9.3	25.8	72.0	46.8	42.5	49.0
Substantive techniques										
Qualify relationship status										
Introduction	5.3		1.3							
Reference to past encounter	8.7		1.3				1.0		1.4	
Reference to future encounter	28.0	3.2	4.0	8.0	3.3	3.0	9.7	3.0	8.2	2.0
Manner of address										
Formal address	94.0			5.3	1.3		0.3			
Nickname or term of endearment			9.3	1.3		1.5	3.0	2.8	11.0	4.0
Emotional expression										
Deference	28.7	3.2	9.3	6.7	2.7		4.0	2.6		6.0
Personal detail	3.3	12.9	2.7	1.3	0.7	0.8	3.3	0.1	4.1	1.0
Gratitude	80.0	6.5	17.3	16.0	17.3	7.6	8.7	1.9	4.1	
Relatability	18.0		13.3	10.7	1.3	1.5	5.0	1.7	9.6	
Well wishes	27.3	12.9	13.3	6.7	4.7	3.8	6.3	1.4	4.1	
Enthusiasm	10.0		13.3	12.0	12.7	4.5	13.3	3.8	5.5	5.0
Empathy	10.0		2.7		1.3	0.8	0.7	1.8	4.1	
Check-in			1.3	4.0	5.3	9.8	9.0	12.4	2.7	8.0
Support		3.2	4.0	1.3	2.7	1.5	10.0	7.0	9.6	9.0
For you. . .			4.0	2.7	4.0	3.0	2.0	0.9	1.4	3.0
"Love"			1.3		21.3	1.5	7.0	0.3	5.5	5.0
Romantic expression									13.7	7.0
Subject matter										
Confirmation or agreement	5.3	9.7	10.7	16.0	6.0	13.6	15.0	11.1	4.1	3.0
Apology	6.7	12.9	9.3	2.7	6.7	4.5	6.3	2.4	4.1	2.0
Excuse	19.3	22.6	17.3	1.3			6.7	4.3		
Humor	6.0		6.7	2.7	20.7	9.1	22.0	7.0	16.4	14.0
Banter					0.7	2.3	3.7	9.8	4.1	17.0
Micro-coordination	1.3	6.5	1.3	5.3	2.0	12.9	2.7	6.8	5.5	3.0
Link	1.3				17.3		11.0	0.4	9.6	
Gossip				1.3	0.7	0.8	5.3	3.2		4.0
Collusion and secrets			1.3		2.7	0.8	6.7	3.9	2.7	2.0
Sexual matter								0.2	16.4	7.0
Derision					0.7	1.5	7.0	5.6	6.8	1.0
Discursive techniques										
Abbreviations			5.3	2.7	3.3		3.0	1.0		1.0
Intentional misspelling			6.7	1.3			9.7	3.2	1.4	
Extra letters	0.7		9.3		7.3	6.1	9.7	4.3	1.4	4.0
All capital letters	0.7		1.3		4.7		9.0	0.4		

(*Continued*)

Table 5. (Continued)

	Authority figures		Peers		Family		Friends		Romantic partners	
	**Email**	**SMS**	**Email**	**SMS**	**Email**	**SMS**	**Email**	**SMS**	**Email**	**SMS**
Emoticon or symbols	0.7		8.0		2.7	3.0	6.7	1.3	6.8	3.0
Social functions										
Establish relationship	4.7			1.3					2.7	
Maintain face	48.7	22.6	29.3	9.3						
Maintain ties	0.7	3.2	1.3	4.0	34.7	15.2	6.7	4.8	5.5	1.0
Increase solidarity	26.7	38.7	10.7	16.0	32.0	15.2	23.0	15.1	15.1	18.0
Mutual activity	2.0				12.7	4.5	3.0	0.4		4.0
Emphasize team membership	5.3		9.3		8.0	6.8	22.7	14.7	6.8	
Confirm intimacy			1.3		16.0	3.0	15.3	13.1	65.8	53.0
N messages	150	31	75	75	150	132	300	900	73	100

Note: All numbers (except *N* messages) are percentages. Cells with no messages are left blank.

https://doi.org/10.1371/journal.pone.0273726.t005

commonly exchanged types of information are excuses (19.3% of emails and 22.6% of texts), apologies (6.7%, 12.9%), and confirmation or agreement (5.3%, 9.7%).

Discursive techniques. Subjects exhibit almost no personal style in communications to authority figures. This highlights a desire to show respect through formal organization and writing structure, like traditional written communication. Even in text messages to peer authority figures, individuals do not use discursive techniques that could compromise a show of professionalism. In other words, even though texting is seen as informal as a rule, subjects still use it in the most distancing way possible.

Social functions. The primary social functions of communications to authority figures are increasing solidarity (26.7% of emails, 38.7% of texts) and maintaining face (48.7%, 22.6%). Subjects used text-based digital communication more to preserve ties than to strengthen them.

Example.

Dear Professor Terrell,

I hope you had a good weekend! I am writing regarding the most recent paper, which is attached. I am really sorry that it is late; I was dealing with a sudden personal crisis and just couldn't finish it by the deadline yesterday. Please do not take this tardiness to be any indication of my commitment to the class, which I am enjoying thoroughly and find really interesting.

Looking forward to lecture tomorrow,

Melanie *(F email to authority)*

[All subsequent sub-sections provide illustrative emails *and* text messages. The infrequency of texts to authority figures makes all such messages atypical and it would be misleading to suggest one as a prototype.]

Peers

Though similarly polite, messages to peers contain a wider array of features than those to authorities and have more personal character. They often contain practical information coupled with attempts to connect with the recipient, such as through gratitude, relatability, or confirmation. Many use corrective face-work—typically excuses rather than apologies. Unlike

with authorities, text messages are employed freely. They serve to maintain face and increase solidarity.

Intended purpose. To peers, participants were most likely to send emails about practical information (66.7%), while texts include both non-practical (38.7%) and practical (32%) information. Interestingly, 26.7% of emails contain corrective face-work, but only 4% of texts. This reinforces the main difference we observed between email and SMS: Because emails are longer, more susceptible to formal organization, and easier to compose because of the full-sized keyboard, they tend to be used in an "official capacity" (Interview 4).

Substantive techniques. When engaging peers, subjects tend not to contextualize the communication. The most frequent form of qualification occurs in texts that reference a future communication (8%). The most commonly expressed emotions are gratitude (17.3% for email, 16% for text), relatability (13.3%, 10.7%), enthusiasm (13.3%, 12%), and well wishes (13.3%, 6.7%). Though choice of medium depends on message purpose—email for administrative and SMS for social tasks—both convey a similar range of emotions. The information most frequently contained in texts is confirmation or agreement (16%), while in emails it is excuses (17.3%). Second most frequent in SMS is micro-coordination (5.3%).

Discursive techniques. Discursive techniques are used slightly more often with peers than with authority figures, particularly in emails. Only 3 texts (4%) contain any discursive techniques, while 23 emails (30.7%) do. In emails, the most common techniques are extra letters (9.3%), emoticons or symbols (8%), and intentional misspellings (6.7%). Still, these are relatively low figures in comparison with messages to family, friends, and romantic partners (below).

Social functions. The most frequent functions of communications with peers are maintaining face (29.3% of emails, 9.3% of texts) and increasing solidarity (10.7%, 16%). That these are the two most common functions of messages to authority figures as well points to the similar role text-based communication serves in these very different types of relationships.

Examples.

> Hey Rachel! Tell everyone sorry I'm not at the meeting—my professor wouldn't stop talking! He just finished. I'll do the slides to make up for it? Let me kno. . . (*M text to peer)*
>
> hey there! id love to talk to you about madrid—youre seriously going to have the BEST semester ever. want to just grab a meal in a dhall [dining hall]? just name a time and place and im there!
>
> talk soon!
>
> christine *(F email to peer)*

Family

Communications to family members are characterized by the exchange of information, both practical and non-practical. They are casual, cheerful, and thoughtful and convey extreme comfort with their recipient. Subjects primarily use text messages to micro-coordinate and confirm or agree and emails to joke and send links. Such communications maintain ties, increase solidarity, and confirm intimacy.

Intended purpose. The most frequent use of digital communications among family is relaying practical information (40.7% of emails, 32.6% of texts), followed by relaying non-practical information (32.7%, 36.4%) and relationship work (9.3%, 25.8%). That these three purposes collectively make up almost all familial communications (88.3%) is notable. The low incidence of arrangement messages may not be generalizable; because our sample is

predominantly composed of students who attend college away from home, it likely reflects their distance from families rather than typical communication habits.

Substantive techniques. Individuals are less likely to qualify their relationship status or use any form of address with family than with anyone else. Because we requested communications with immediate family members only, these data reflect recipients with whom subjects most likely have frequent and longstanding interactions. This, combined with a high level of intimacy, means contextualization is generally unnecessary. The most frequently expressed emotions are gratitude (17.3% of emails, 7.6% of texts), enthusiasm (12.7%, 4.5%), and love (21.3%, 1.5%). In all, there are more emotional expressions in emails—likely due to the length restrictions of SMS—but more check-ins in text messages—because such messages are short and intended to be immediately received.

The type of information exchanged with family is more dependent on medium than the two previous relationship categories. In email, most frequent are humor (20.7%) and links (17.3%)—and then, with a substantial drop, apologies (6.7%) and confirmation or agreement (6%). In texts, most common are confirmation or agreement (13.6%), micro-coordination (12.9%), and humor (9.1%). Humor is used with family more than any other relationship besides friendships, reflecting an overriding geniality to these communications.

Discursive techniques. Participants employ more discursive techniques with family than with peers or authority figures. This is representative of their generally increased emotionality compared to the previous, more socially distant categories of recipients. Using extra letters is the most widely applied technique, occurring in 7.3% of emails and 6.1% of texts.

Social functions. Communications with family serve two general functions: maintaining ties (34.7% of emails, 15.2% of texts) and increasing solidarity (32%, 15.2%). Emails also confirm intimacy (16%) and engage cointeractants in mutual activity (12.7%). This certainly relates to the high level of non-practical information families exchange; links, too, provide subject matter for jocular discussion. By keeping family members tuned in to their daily lives and sustaining casual dialogue, subjects accomplish emotional closeness despite physical distance.

Examples.

hayyy im heere where are you? (F *text to family)*

Subject: Self-Righteous Michael Kubin Editorial

http://www.nytimes.com/2010/12/12/opinion/12kubin.html?scp=1&sq=michael%20kubin&st=cse

Don't know if you guys saw this. Thought you may enjoy. Denis *(M email to family)*

Friends

Respondents text and email friends persistently throughout the day; these exchanges are arguably as integral to friendships as face-to-face interaction. Unlike previous categories of relationships, these messages primarily concern recreational plans and friendship work. This is accomplished through high rates of emotional expression, including support, check-ins, gratitude, love, and frequent use of humor. Banter and link-sending are common. Between friends, digital interaction heightens solidarity, affirms team membership, and confirms intimacy. Though we separately requested communications to same- and opposite-sex friends, there were few systematic differences in the features of each; we therefore treat them here as one category.

Intended purpose. Communications among friends are highly intimate. Practical information is still prevalent (22.3% of emails, 9.3% of texts), but social arrangements (11.3%,

10.2%) and relationship work (72%, 46.8%) are more common here than for any other relationship type. The heavy reliance on these digital mediums for friendship work shows they have been fully integrated into the fabric of daily communications among strong ties. Moreover, the difference in non-practical information exchange between friends (21.3%, 16.9%) and family (32.7%, 36.4%) suggests texts and emails are more integral to the day-to-day *execution* of friendships—as opposed to keeping family informed about one's life.

Substantive techniques. Friendships are qualified infrequently and typically in reference to a future communication (9.7% of emails, 3% of texts). Similarly, subjects seldom directly address the recipient, except with a nickname or term of endearment (3%, 2.8%). Emotions expressed toward friends depend on the medium: Subjects expressed more gratitude (8.7% vs. 1.9%), enthusiasm (13.3%, 3.8%), support (10%, 7%), and "love" (7%, 0.3%) in emails but "checked-in" more frequently via SMS (9%, 12.4%). The most frequent type of information is confirmation or agreement (15% of emails, 11.1% of texts) and humor is more prevalent than for any other relationship type (22%, 7%). Also relatively common are micro-coordination (2.7%, 6.8%), link-sending (11%, 0.4%), and text message banter (9.8%). Only in messages among friends are derision (7%, 5.6%), gossip (5.3%, 3.2%), and collusion or secrets (6.7%, 3.9%) notably present. These data strongly suggest that friends' interaction is fairly sustained, providing ongoing context for digital exchange. Meanwhile, the more fragmented nature of encounters with family would explain the greater need for contextualization, most easily accomplished within the affordances of unlimited email length.

Discursive techniques. Of all types of relationships, individuals are most likely to use discursive techniques in messages to friends. Most frequent are extra letters (9.7% of emails, 4.3% of texts) and intentional misspellings (9.7%, 3.2%)—again pointing to the casualness of these exchanges and the informal tone participants create. We attribute the disparity in these techniques between email and SMS to interface: Most are easier to accomplish on a keyboard.

Social functions. Through digital messages, friends confirm intimacy (15.3% of emails, 13.1% of texts), emphasize team membership (22.7%, 14.7%), and increase solidarity (23%, 15.1%)—functions that deepen socio-emotional bonds. Gossip and derision validate the divide between "us" and "them." Confiding in a friend expresses trust and further strengthens the relationship through mutual preservation of a secret. Furthermore, by showing recipients how they are like-minded with the sender, high rates of confirmation continuously heighten solidarity.

Examples.

> hiiiii! how are you lover?? we should talky soon miss you kiss you *(F text to friend)*

> haha true good point, but i was dying when i read it [www.whenparentstext.com]. and the eighth down. i've got to stop looking at this site and write these goddamn papers, shitttttttt *(M email to friend)*

Romantic partners

Similar to friends, partners use text messages and email to stay in touch throughout the day, engaging in sustained romantic relationship work and banter rather than achieving practical ends. Communications are characterized by overt expressions of affection and a cheerful tone; they are positive and warmhearted. Partners seldom employ discursive techniques. Rather, messages check-in, convey support, and often contain humor. They increase solidarity and confirm intimacy.

As with some other categories of relationships, our data on romantic partners are incomplete. Of the 15 total participants, only five females and five males had someone they would

consider a partner. While all ten provided the full ten SMS messages we requested, only eight (four women and four men) exchanged emails with their partner, and one of these women had sent only three. As the male with no romantic emails explained, "I'm always just texting with girls. I really only send emails if I have to; emailing a girl would be weird. The only girl I email is my mom" (Interview 3).

Intended purpose. The primary purpose of messages between partners is relationship work (42.5% of emails, 49% of texts), followed by relaying non-practical information (35.6%, 17%). In other words, they communicate for the sake of communication and to remind each other that they care. Social arrangements make up only 11% of emails and 12% of texts, suggesting such arrangements are made in other ways—presumably in person. Before digital technology, such a large amount of relationship work would require being on the phone or physically present with one's partner. Now, couples can engage in this work more often and while doing other things like sitting in class, hanging out with friends, or working—all completely isolated from one another [89].

Substantive techniques. Similar to other intimate ties, participants rarely qualify their romantic partnerships. Though occasionally they call partners by nicknames or terms of endearment (11% of emails, 4% of texts), this is surprisingly uncommon. The most frequent information types in email are humor and sexual subjects (both 16.4%), whereas in text messages banter (17%) is most common—painting a lighthearted portrait of digital exchange. The most frequent emotion in emails is romantic expression (13.7%) and in texts, support (9%). Unsurprisingly, sexual subjects and romantic expression were more common among partners than all other relationship types.

Discursive techniques. Participants used few discursive techniques with their partners; only 9.6% of emails and 8% of texts contained any. However, emoticons are used more frequently between partners than any other relationship type. Because these communications are already so light-hearted and relation-oriented—and because partners are more comfortable expressing emotions explicitly—much of the need to rely on discursive (as opposed to substantive) devices may be eliminated.

Social functions. Over half of messages between partners confirm intimacy (65.8% of emails, 53% of texts). As evidenced by the distribution of purposes, participants use digital communication to stay close to their partners throughout the day. Through notes of affection and humor, they maintain the strength of their connection even while apart. These messages also increase solidarity (15.1%, 18%); by expressing agreement, partners affirm elements of a shared social consciousness.

Examples.

> ugh it's pouring! guess the gloomy weather means we have to snuggle all afternoon. . . bummerrrrr: D call me when you're out *(M text to romantic)*
>
> Hellooo pup. Sorry we didn't get a chance to talk this morning. Currently am in the car on my way to polo. Miss you very much and can't wait to talk to you tonight! Have a great day. Hugs and kisses
>
> also: http://5napkinburger.com/ coming to boston (!!!)
>
> (F email to romantic)

Discussion

In this section, we consider the implications of our results in terms of the nature, relational patterning, and societal impact of text-based digital interaction. Some of the points we make

have received little or no prior attention; others support, confirm, or expand upon findings from prior research.

Dramaturgy in the digital age

In the voluminous literature on CMC, many others have commented on the distinct nature of digital (as opposed to face-to-face) exchange. One challenge of this research is that "digital" takes so many forms, creating obstacles to triangulation. Particularly notable in this regard are social media [50, 51], where the richness of interaction possibilities on any one platform—and tremendous variation across them—make precise characterizations of any one "medium" all but impossible. Further complicating matters is that many such sites no longer exist (or have changed dramatically over their lifespan), making it difficult to pinpoint how each functioned, when. In contrast, our focus on email and SMS helps clarify which findings may be unique to certain platforms or potentially innate to text-based exchange. Like many scholars before us, we find dramaturgy useful as an organizing lens.

An elevated personal front. According to Goffman [36], front is comprised of a performer's 1) setting and 2) personal front, or the self-image she projects. Because there is no physical setting in digital exchange, the role of the personal front is substantially elevated. At the same time, many of its features—including body language, facial expressions, and current appearance—are unknown. Thus, it is at once interactants' *only* source of information and depleted of many of its detail-providing capabilities [28].

The consequence of this shift is that manner is reduced to explicit expressions. Face-to-face manner is frequently unintentional: Someone is excited, so her eyes light up. While such mannerisms are often strategic—like staring at a blackboard while thinking about the weekend—they are always deliberate in digital exchange. For instance, a digital interactant might write, "Yayayayayayayay OMG OMG I LOVE YOUUUU" (DT.90). Here, traditionally tacit information (great enthusiasm and friendliness) is shared through intentional decisions (capitalization and extra letters). Impressions "given off" are thus in fact impressions "given" because all parts of a presentation are consciously chosen [26, 34].

Two implications are notable. First, because digital personal fronts are so important—and presumably deliberate—instances when rules *about* them are violated may be particularly jarring. Nervous laughter can't be helped, but explicitly typed laughter ("hahaha") at an inappropriate time could seem offensive. Similarly, just as it would be strange to yell a private matter, communicating sadness in all capital letters would (almost humorously) undermine the performance of sorrow. Successful face-to-face performances require that appearance, manner, and setting are all congruent. It is equally, if not more essential to digitally communicate with consonant substantive and discursive techniques—reflected in the great care with which messages (especially to authorities) were constructed and even "proofread" (Interview 2).

Second, however, some people may *prefer* this explicit articulation of meaning under challenging or uncomfortable circumstances like corrective face-work [112]. On one hand, apologies (or excuses) can be carefully crafted to contain the right tone and detail (e.g. IP.15 and the illustrative message provided at the end of the section on authority figures). On the other hand, one can avoid the emotions face-to-face interaction may evoke—such as the embarrassment of looking someone in the eye following a role violation—and the challenges of both verbally and non-verbally performing remorse. As one respondent summarized, "It's easier to say how you're feeling when the person is not sitting right in front of you" (Interview 8). The physical distance and temporal flexibility of textual interaction may be advantageous in this case, insofar as individuals are more likely to apologize. Similarly, another participant expressed a preference for *receiving* unexpected or potentially challenging communications this way: "Say

I hurt someone's feelings, I would rather them text me so I can figure out what I did wrong and be prepared to apologize, rather than if someone is visibly upset and I'm caught off guard" (Interview 10).

Greater temporal complexity. Because email and SMS are asynchronous, time is important beyond the nuances of individual mannerisms [58]. While face-to-face exchanges have their own temporal rhythms [113], pauses are generally short and *some* response can be assumed. In contrast, digital response times vary widely—from moments to minutes to months—and can be motivated by a wide variety of intentions or circumstances—from being in class with your phone on "silent" to intentionally ignoring a message. Consequently, while substantive and discursive decisions are viewed as deliberate, the issue of whether or how time is meaningful is a matter of constant ambiguity.

Exemplifying this is when a response is absent or substantially delayed. Although short response times are typical among intimate ties, it is universally understood that people may take a while to respond. (There is also a general leniency awarded to authority figures. One participant accounted for this with the power dynamic between professors and students, bemoaning, "You are beholden to them so you can't complain"; Interview 14.) However, during rapid exchanges—of texts or emails—a sudden change of pace may be interpreted to reflect frustration or anger. As one participant elaborated, "I mean the person on the other end clearly asked a question or said something to you. And it's like, 'I clearly read this because I always have my phone and I'm not responding.' It's pretty obvious they're pissed" (Interview 2). In this way, such delays are not only symptomatic of becoming angry and not wanting to talk any longer—they are established non-verbal mechanisms of *communicating* these emotions.

And yet, long response times can mean many other things. They can be attempts to seem cool and easy-going, particularly when people are flirting or just becoming friends: Though the recipient may anxiously await each message, a delayed response attempts to conceal this fact—giving a relaxed or indifferent impression (Interview 4). They can also be unintentional, such as when an interactant is interrupted by someone in person. In this sense, it is striking how *accurately* participants interpret delays based on past information and situational cues; physical separation hardly prevents keen attunement to moods and communication patterns among intimates.

An expanded backstage region. With the transcendence of physical space comes a fundamental redefinition of performance regions. Because a digital message can be composed from anywhere (and typically out of sight of the audience), the entire performance is *created* and *remains* backstage (in the sense of a physical space where one can engage in discordant behavior) until the moment the "send" button is tapped. While this enables a variety of situations impossible offline—such as instantly jumping between roles in different messages—we focus on two particularly important implications:

First, actors have increased *resources* at their disposal—including not only time [114], but anything available in their current setting that could aid the projection of front, in ways face-to-face presence could never allow. Several subjects confessed to consulting dictionaries, newspapers, and websites when writing messages. The potential tools for digital presentation of self are virtually limitless and extend far beyond one's own knowledge and front-repertoire—including the knowledge and repertoires of other people.

Second, then, the redefinition of regional boundaries creates new possibilities for *team* collaboration. Every participant in our study admitted to sharing emails or texts with friends and both giving and receiving advice on how to respond. One message can have multiple authors (and multiple audiences—see below). Consequently, teams can collaborate not only to preserve a group's image, but also the image of a single person. Another new possibility is discreet communication in the presence of others. Face-to-face collusion is often tacit, as team

members work off one another but can only openly discuss their line beforehand or afterwards. In digital interaction, actors can discuss and defy their performance in the very act of performing it, such as the erotic text sent "at work" in ST.59.

Portable, permanent performances. While the backstage region expands, the front stage region shrinks *and* expands in digital interaction. On one hand, the recipients of emails and text messages are deliberately chosen (whereas in person, it is not always possible to isolate one's intended audience). In this way, digital interaction is highly privatized. On the other hand, offline performances are limited to the space and time in which they occur. Digital performances, in contrast, are effectively fossilized: Once they have been "performed" (i.e. sent), they exist *independently* of the performer—who loses all control over (and awareness of) potential audience members. Further, messages can theoretically exist forever and are easily disseminated [112]. In other words, unlike face-to-face performances—which tend to be stationary, temporary, and limited in scale by space constraints—digital performances are portable, permanent, and potentially public (Fig 1).

This results in a new and more self-aware type of performance. While performers present a front that is appropriate for the current message, they often do so in a manner that would not threaten their face in other relationships *should* the exchange be shared. In ST.71, for example, the participant's mockery of his cousin is implicit—he does not state that he is sending the link at his cousin's expense. In ST.73, though the interactants talk about a girl, they are careful never to state her name should someone outside their circle happen to see the communication. Explained one subject, "I try not to say people's names if we're talking about them [in text messages] because I'm paranoid I'm going to lose my phone" (Interview 12). Other interviewees reported experiences of disaster when past digital conversations were made public (Interviews 6, 7, 11). Whereas embarrassment in the present is a policing factor in face-to-face interaction, the potential for future embarrassment restrains digital exchange.

We contrast these dynamics with "context collapse" [45], where differentiation in self-presentation is impossible because multiple audiences are flattened into one. Davis and Jurgenson's [115] concept of "context collision"—the *unintentional* merging of audiences—may be more relevant. Meanwhile, these findings are otherwise similar to boyd's [116] characterization of social media (especially her concepts of "persistence," "scalability," and "invisible audiences"), underscoring the importance of differentiating between general properties of "bit-based content" (p. 46) and the specific *manifestation* of these traits in "networked publics" [117].

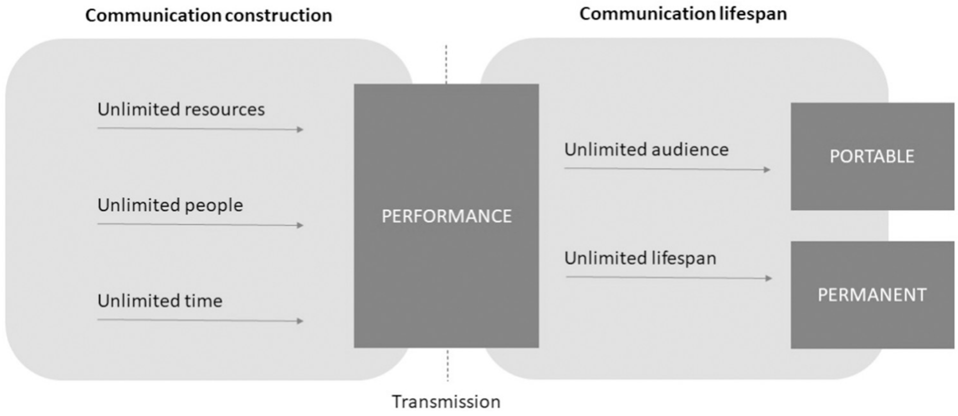

Fig 1. Portable and permanent performances. Individuals have unlimited time and resources at their disposal—including collaboration with team members—to craft a digital message. Once that message is sent, however, it becomes fossilized and the recipient may easily share it with anyone without the sender's knowledge or consent.

https://doi.org/10.1371/journal.pone.0273726.g001

The relational patterning of digital communication

Emails and text messages are used by people of all ages and backgrounds. A basic feature of social life is that the substance of communication is tailored to the audience at hand. However, *how* the properties of digital interaction vary by relationship type has received little systematic attention [89].

While our data also showed important variation by medium, we were surprised to find it was not more pronounced. Further, such differences stemmed primarily from technological constraints: Compared to emails, texts convey less information due to character limits, lack space for conventional organization, and are harder to compose; consequently, they are more likely to be used when information is simple and users desire a fast response. Importantly, however—and plausibly due to these traits—SMS is also viewed as more casual [118]. When asked why they seldom texted authority figures, participants did not cite inability (though presumably few had access to the cell numbers of their professors or TAs). Instead, they indicated it would be "too informal" (Interview 1) or just felt "wrong" (Interview 8).

This qualitative impression (that the medium is less determinative than the relationship) was supported by a supplementary quantitative analysis performed using UCINET [119]. Specifically, we considered the summary statistics presented in Table 5—a matrix of 47 codes (rows) by 10 relationship types (columns)—as the raw data for a cluster analysis. First, we calculated the distance between each pair of relationship types as the Euclidean distance between the relevant two vectors of code frequencies (measured as percentages). This generated a 10-by-10 matrix of dissimilarity scores. (Alternative measures of distance produced similar results.) Second, we ran a hierarchical clustering algorithm on these dissimilarity scores based on a weighted average approach, i.e. where the distance between clusters is the average dissimilarity value weighted by cluster size. The dendrogram from this analysis (Fig 2) shows that

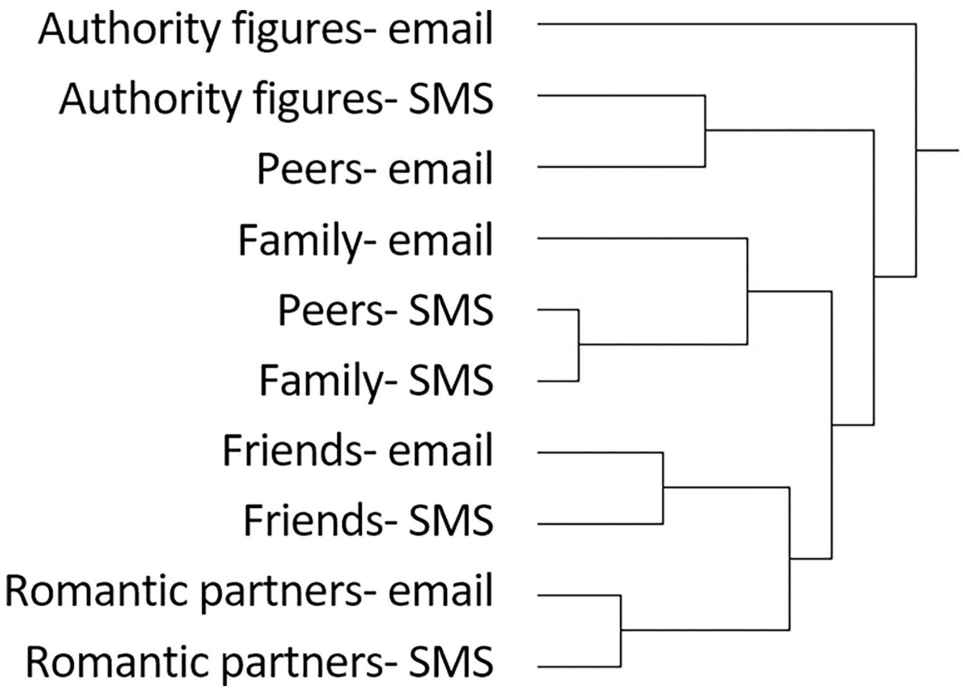

Fig 2. Results from hierarchical cluster analysis of communication types based on data in Table 5. The dissimilarity among types was measured using Euclidean distance and the clustering solution was produced using a weighted average approach. Analysis was performed using UCINET [119].

https://doi.org/10.1371/journal.pone.0273726.g002

based on the distributions of codes, communications sent to the same category of recipient are consistently more similar than communications sent using the same medium.

To our knowledge, this *similarity* between texts and emails—conditional on the nature of the relationship—has not before been identified, leading to a potential over-emphasis in the literature on the distinctiveness of each. In fact, among all 47 major and minor codes we identified, only two ("introduction" and "reference to past encounter") were unique to a certain medium (email).

While communications in each relationship category were characterized by a unique constellation of traits, the primary schism we observed was based on the *intimacy* of the relationship—where messages to family, friends, and partners (i.e. more intimate ties) were generally similar, as were messages to peers and authority figures (i.e. less intimate ties). Communications with authorities show respect through deference, gratitude, and formal address and primarily function to heighten solidarity and maintain face. Digital interactions with peers are most comparable, particularly with respect to their functions and formal style. In contrast, communications among intimates are lighthearted, loving, and supportive; they perform relationship work and confirm intimacy; and discursive techniques are common.

Within these two groups—low intimacy and high intimacy—the element most predictive of variation in message properties is *age*. Specifically, when communicating with peers as opposed to authority figures, or friends and partners as opposed to parents (the majority of recipients in the "family" category), subjects are engaging fellow members of a generation characterized by strong digital fluency—people who share a nuanced technological and cultural understanding of "how" to use modern devices. Accordingly, messages to bosses the same age as respondents looked very similar to messages to peers.

Finally, though digital communication transcends space, the *physical distance* between interactants still impacts the form messages take [120]—though our data suggest it is the weakest predictor of the three. In contrast, Johnson et al. [73] found little impact of distance on college students' relationship maintenance strategies; Boneva et al. [17] found men and women have different patterns of long-distance communication; and Mok et al. [96] found frequency of contact is generally insensitive to distance, except for transoceanic relationships. (All three studies examine email, not SMS.) While all five of our relationship types can be generally classified by intimacy and age, the distinction between proximate and distant relations arose inductively. Of course, proximity is likely correlated with other variables (such as how frequently the sender and recipient tend to see one another); accordingly, some message features (e.g. practical arrangement) are less relevant for distant ties.

Moving from the most foretelling property (intimacy) to the least (physical distance), it also appears that a "distant" value on any one relational component limits the predictive power of those that follow. For example, if the sender and recipient share a casual relationship, age has limited impact and the influence of physical distance is negligible. If the relationship is intimate, however, age increases in importance: A text message to a parent may differ markedly from one to a sibling. Lastly, proximity matters most for intimate, same-age ties, and has less impact on messages to older recipients (Fig 3).

Digital exchanges and modern society

Lastly, we consider potential byproducts of the widespread use of SMS and email on select features of everyday interaction and on the structure of social networks.

Sociability, stability, and solidarity. Although digital technology has transformed social life in many ways, we emphasize three consequences implied by our data. First, the accessibility of digital communication undeniably impacts the frequency of certain forms of interaction —making some, like sociability [121], easier to perform. Today, if someone is bored (e.g.

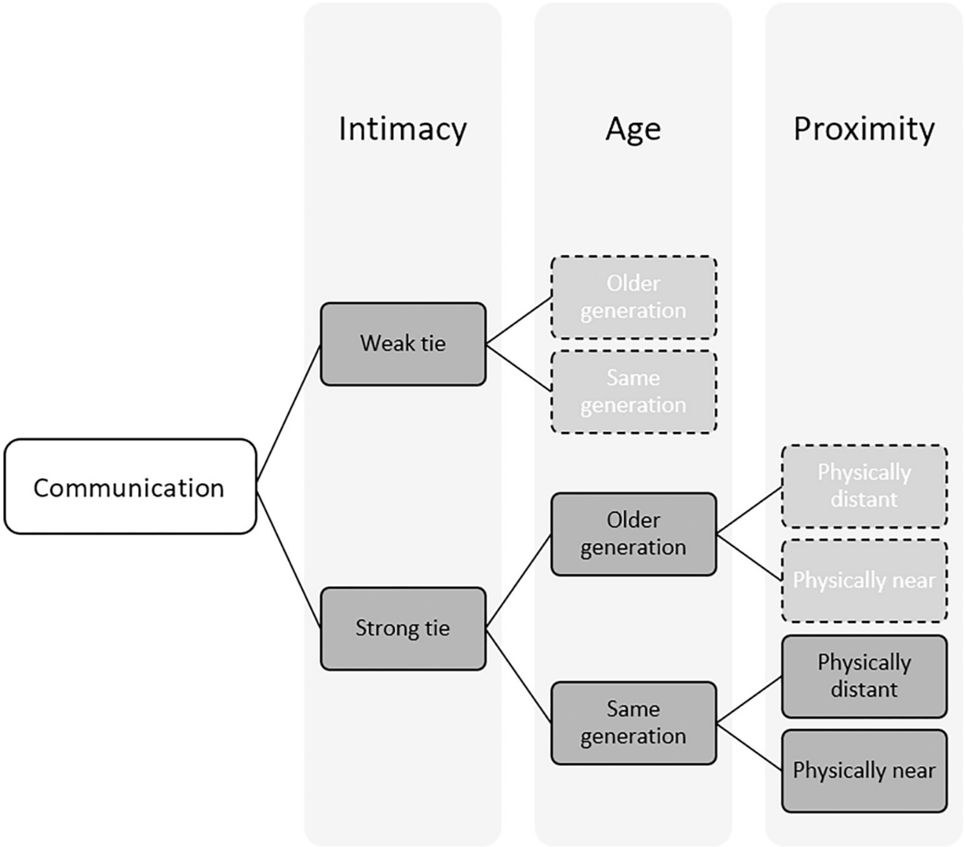

Fig 3. The predictive importance of intimacy, age, and proximity. The influence of a relationship feature on the structure of a communication decreases the further to the right and the more transparent that feature appears in the above diagram. Thus, opaque features are more important and transparent features less so; and intimacy is more important than age, which is more important than proximity.

https://doi.org/10.1371/journal.pone.0273726.g003

during "idle times" like riding public transit [60, 122]), she can text a friend to pass the time (not unlike listening to a Walkman or playing a Game Boy not long ago—functions also absorbed by contemporary smartphones). Digital communication facilitates communication for communication's sake; it is out of this utilization of texting, for instance, that banter like ST.66 and ST.74 are born and (within our "intended purpose" family of codes) non-practical information is exchanged.

Second, however, this ease of access has its costs. A central example is micro-coordination. As a rule, it is inessential—it is a tool of politeness and convenience borne of technological capacity. But because cointeractants can always be reached, plans are always subject to last-minute change; the availability of digital communication thus serves to both confirm *and* undermine plans until the very moment of their execution, decreasing the stability of arrangements [123].

Third, the theme throughout our data of shared sentiment in social groups naturally invites reference to mechanical solidarity [124, 125]. Explicitly captured by our "increase solidarity" code, digital channels facilitate information diffusion and shared knowledge; exchanging links, pictures, and jokes elicits common emotional reactions, magnifying feelings of closeness; and shared distaste towards others (e.g. gossip or derision) further affirms the consciousness of the in-group. That email and SMS are more often used to emphasize similarity rather than difference could also reflect the constraints of text-based exchange (where nuance is inconvenient,

emotional monitoring is difficult, and odds of misinterpretation are high—unfortunate staples of online debate that close ties might prefer to avoid).

Broader and deeper social networks. Digital exchange has low costs and high returns. It does not take much time to craft a short message, yet such basic acts of communication (from well wishes to romantic expressions, check-ins to "for yous. . .") enrich and revitalize social ties [89, 97]. This important "social function" was also explicitly identified by our coding. On one hand, the widespread use of SMS and email make it incredibly easy to communicate with people we are unwilling or unable to contact by traditional means—including introductions (e.g. ST.28, ST.42, and Interviews 2 and 12). On the other hand, young adults today are saturated with an endless flow of messages from family, romantic partners, and friends, even when they are physically distant—maintaining high levels of intimacy that might have faded in a previous age [8, 22]. Consequently, digital interaction facilitates both the maintenance of weak ties outside our normal social circles as well as the frequent activation of strong ties near or far [89], effectively "broadening" and "deepening" our social networks (Fig 4).

These changes entail benefits and challenges alike. A large body of literature documents the advantages of CMC, including increased authenticity and self-disclosure [26, 126, 127]. Digital interaction can also facilitate other forms of exchange: Friends can be invited to join someone at a bar (IP.21); plans can be made to catch up by phone (IP.24) or other "logistics" can be arranged (IP.1-6); and because individuals do not have to reestablish lines when they have stayed in touch digitally, face-to-face interactions are smoother. Indeed, the more time that has passed between offline encounters, the larger the possible gulf between performances—and the greater the utility of digital exchange for bridging this fracture.

But there are limits to network engagement—even digitally [128]. In a sense, young adults are so frequently in touch because there is no excuse *not* to be. However, such communication detracts from other experiences—including private activities, offline interaction, and valuable (but increasingly scarce) moments without external stimulation [129]. Many relationships now subsist on validation in a way they could not, previously. Rather than unspoken and enduring bonds, such ties must be constantly tended to avoid disintegration—a delicate (and potentially draining) endeavor.

Conclusion

Digital communication is now so accessible and widespread that any portrait of modern social life seems incomplete without it. This is particularly true for young adults: 48% of 18- to

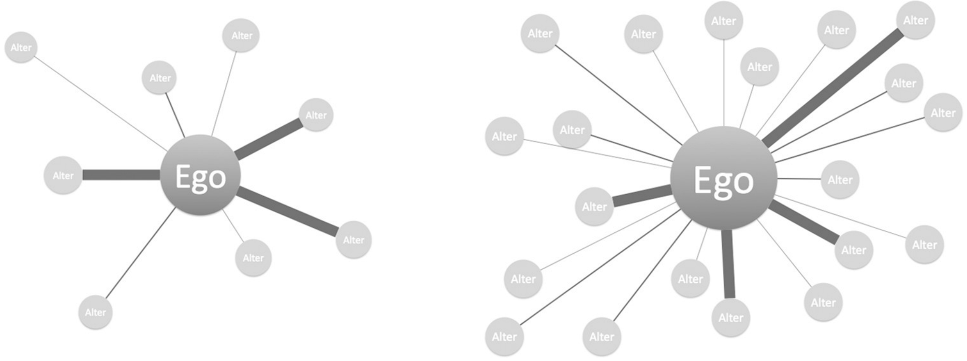

Fig 4. Broader and deeper social networks. Digital communication may alter the structure of social networks (from left to right, above) by making it easier to maintain weak ties (thin connections) and strong ties (thick connections) alike.

https://doi.org/10.1371/journal.pone.0273726.g004

29-year-olds say they go online "almost constantly" [130]. On one hand, the popularization of the smartphone—which makes email immediately accessible—and especially the iPhone—which enables "text messages" (via iMessage) to surpass 160 characters—has made emails and texts increasingly indistinguishable, potentially blurring the differences we observed. On the other hand, these mediums are now richer than ever before (including not just pictures and animations, but meta-communication such as "likes" and "laughter"); additional features (such as read receipts and "typing. . ." notifications) further complicate the temporal dynamics described above; and the plethora of available platforms (from Instagram to Facebook, WhatsApp to Zoom) mean that emails and text messages are but two options on an ever-diversifying communicative menu. For all of these reasons, it is that much more important to have an analytic framework for comprehension and an empirical baseline for comparison.

To what extent did we achieve these ends? Given the peculiarities of our research design, it seems appropriate in closing to return to the core issues of generalizability our data raise: namely, the extent to which self-selected, text-based messages furnished by a convenience sample of college students in 2010 are relevant to a broader population of people and the many digital interaction forms available today.

First, we believe our research would not be possible with a random sample of participants or messages; indeed, that *anyone* was willing to provide such an intimate look at their private lives was fortuitous (and our response rate was still only 65% among initial contacts). Though supplied by 15 respondents, our vast dataset of messages generated a nuanced and transposable theoretical framework that meets evaluative standards for our method [100, 131]. Comparisons by medium and relationship type established vibrant empirical patterns, even if precise code distributions are ungeneralizable. Future research could identify additional codes our research design precluded; examine other kinds of relationships beyond (or within) the five examined here (for instance, colleagues, adversaries, or partners of varying duration or commitment); or even use our results to seed automated analyses of large corpora without examining individual messages, simultaneously protecting author privacy and enhancing scale [132].

Second, our methods have enabled us to show that some results produced by studies of specific digital platforms (often social media) are in fact generic, helping bridge the gap between broad, classic theories of computer-mediated interaction [26, 27] and empirical work. Insofar as *all* communication contains content, our framework should be broadly useful—although non-textual and/or synchronous forms may suggest entirely new analytic properties. As digital interaction continues to evolve—replicating, if not surpassing, more features of face-to-face exchange—it will be increasingly valuable to identify precisely which interactional factors are responsible for what substantive consequences. At the outset of this paper, we suggested some basic dimensions of variability, including communicative affordances (e.g. textual vs. audiovisual, synchronous vs. asynchronous), social structure (e.g. dyadic vs. group, familiar vs. anonymous), and technical interface (in terms of ease, accessibility, and length). While other dimensions are possible—including how each manifests in distinct cultural and historical settings—we suggest that any such typology of digital forms might usefully begin with the elementary.

Ironically, given the explosion of "big data" research examining large social systems in microscopic detail [133], digital scholarship has yet to fulfill one of its most enticing possibilities: providing new insight into the *content* of everyday exchanges [134]. Despite its limitations, we hope our work will be perceived as an important and timely step in this direction.

Supporting information

S1 File. Appendices.
(PDF)

Author Contributions

Conceptualization: Lauren S. Brown, Kevin Lewis.

Data curation: Lauren S. Brown.

Formal analysis: Lauren S. Brown.

Investigation: Lauren S. Brown.

Methodology: Lauren S. Brown, Kevin Lewis.

Supervision: Kevin Lewis.

Visualization: Lauren S. Brown.

Writing – original draft: Lauren S. Brown.

Writing – review & editing: Lauren S. Brown, Kevin Lewis.

References

1. Pew Research Center. Internet/broadband fact sheet. Washington, DC: Pew Research Center, 2019.
2. Pew Research Center. Mobile fact sheet. Washington, DC: Pew Research Center, 2019.
3. Perrin A, Anderson M. Share of U.S. adults using social media, including Facebook, is mostly unchanged since 2018. Washington, DC: Pew Research Center, 2019.
4. Golder SA, Wilkinson DM, Huberman BA. Rhythms of social interaction: Messaging within a massive online network. In: Steinfield C, Pentland BT, Ackerman M, Contractor N, editors. Communities and technologies 2007: Proceedings of the third communities and technologies conference, Michigan State University, 2007. London: Springer; 2007. p. 41–66.
5. Kossinets G, Watts DJ. Empirical analysis of an evolving social network. Science. 2006; 311:88–90. https://doi.org/10.1126/science.1116869 PMID: 16400149
6. Eagle N, Pentland AS, Lazer D. Inferring friendship network structure by using mobile phone data. Proceedings of the National Academy of Sciences of the United States of America. 2009; 106:15274–8. https://doi.org/10.1073/pnas.0900282106 PMID: 19706491
7. Adamic LA, Adar E. Friends and neighbors on the Web. Social Networks. 2003; 25:211–30.
8. Ellison NB, Steinfield C, Lampe C. The benefits of Facebook "friends:" Social capital and college students' use of online social network sites. Journal of Computer-Mediated Communication. 2007; 12:1143–68.
9. boyd d. It's complicated: The social lives of networked teens. New Haven: Yale University Press; 2014.
10. Madianou M, Miller D. Migration and new media: Transnational families and polymedia. London: Routledge; 2012.
11. Hargittai E. Whose space? Differences among users and non-users of social network sites. Journal of Computer-Mediated Communication. 2008; 13:276–97.
12. Stearns E, Buchmann C, Bonneau K. Interracial friendships in the transition to college: Do birds of a feather flock together once they leave the nest? Sociology of Education. 2009; 82:173–95.
13. Hays RB, Oxley D. Social network development and functioning during a life transition. Journal of Personality and Social Psychology. 1986; 50:305–13. https://doi.org/10.1037//0022-3514.50.2.305 PMID: 3701579
14. Palfrey J, Gasser U. Born digital: Understanding the first generation of digital natives. New York: Basic Books; 2008.
15. Zickuhr K. Generations 2010. Washington, D.C.: Pew Research Center, 2010.
16. Lenhart A. Cell phones and American adults. Washington, D.C.: Pew Research Center, 2010.
17. Boneva B, Kraut R, Frohlich D. Using e-mail for personal relationships: The difference gender makes. American Behavioral Scientist. 2001; 45:530–49.
18. Thurlow C. Generation txt? The sociolinguistics of young people's text-messaging. Discourse Analysis Online. 2003.
19. Durkheim E. The elementary forms of religious life. New York: The Free Press; [1912] 1995.

20. Petersen MN, Harrison K, Raun T, Andreassen R. Introduction: Mediated intimacies. In: Andreassen R, Petersen MN, Harrison K, Raun T, editors. Mediated intimacies: Connectivities, relationalities and proximities. London: Routledge; 2018. p. 1–16.
21. Silverstone R. Why study the media. London: SAGE; 1999.
22. Chambers D. Social media and personal relationships: Online intimacies and networked friendship. London: Palgrave Macmillan; 2013.
23. Attwood F, Hakim J, Winch A. Mediated intimacies: Bodies, technologies and relationships. Journal of Gender Studies. 2017; 26:249–53.
24. Barker M-J, Gill R, Harvey L. Mediated intimacy: Sex advice in media culture. Cambridge: Polity; 2018.
25. Donath J, boyd d. Public displays of connection. BT Technology Journal. 2004; 22:71–82.
26. Walther JB. Computer-mediated communication: Impersonal, interpersonal, and hyperpersonal interaction. Communication Research. 1996; 23:3–43.
27. Walther JB. Interpersonal effects in computer-mediated interaction: A relational perspective. Communication Research. 1992; 19:52–90.
28. Valkenburg PM, Peter J, Walther JB. Media effects: Theory and research. Annual Review of Psychology. 2016; 67:315–38. https://doi.org/10.1146/annurev-psych-122414-033608 PMID: 26331344
29. Daft RL, Lengel RH. Organizational information requirements, media richness and structural design. Management Science. 1986; 32:554–71.
30. Short J, Williams E, Christie B. The social psychology of telecommunications. London: Wiley; 1976.
31. Sproull L, Kiesler S. Reducing social context cues: Electronic mail in organizational communication. Management Science. 1986; 32:1492–512.
32. Tidwell LC, Walther JB. Computer-mediated communication effects on disclosure, impressions, and interpersonal evaluations: Getting to know one another a bit at a time. Human Communication Research. 2002; 28:317–48.
33. Walther JB, Loh T, Granka L. Let me count the ways: The interchange of verbal and nonverbal cues in computer-mediated and face-to-face affinity. Journal of Language and Social Psychology. 2005; 24:36–65.
34. Walther JB. Selective self-presentation in computer-mediated communication: Hyperpersonal dimensions of technology, language, and cognition. Computers in Human Behavior. 2007; 23:2538–57.
35. Walther JB. Relational aspects of computer-mediated communication: Experimental observations over time. Organization Science. 1995; 6:186–203.
36. Goffman E. The Presentation of Self in Everyday Life. New York: Doubleday; 1959.
37. Robinson L. The cyberself: The self-ing project goes online, symbolic interaction in the digital age. New Media & Society. 2007; 9:93–110.
38. Rettie R. Mobile phone communication: Extending Goffman to mediated interaction. Sociology. 2009; 43:421–38.
39. Humphreys L. Cellphones in public: Social interactions in a wireless era. New Media & Society. 2005; 7:810–33.
40. Papacharissi Z. The presentation of self in virtual life: Characteristics of personal home pages. Journalism & Mass Communication Quarterly. 2002; 79:643–60.
41. Dominick JR. Who do you think you are? Personal home pages and self-presentation on the world wide web. Journalism & Mass Communication Quarterly. 1999; 76:646–58.
42. Krämer NC, Winter S. Impression management 2.0: The relationship of self-esteem, extraversion, self-efficacy, and self-presentation within social networking sites. Journal of Media Psychology. 2008; 20:106–16.
43. Rui J, Stefanone MA. Strategic self-presentation online: A cross-cultural study. Computers in Human Behavior. 2013; 29:110–8.
44. Ellison N, Heino R, Gibbs J. Managing impressions online: Self-presentation processes in the online dating environment. Journal of Computer-Mediated Communication. 2006; 11:415–41.
45. Marwick AE, boyd d. I tweet honestly, I tweet passionately: Twitter users, context collapse, and the imagined audience. New Media & Society. 2010; 13:114–33.
46. Schwartz R, Halegoua GR. The spatial self: Location-based identity performance on social media. New Media & Society. 2015; 17:1643–60.
47. Thumim N. Self-representation and digital culture. Basingstoke: Palgrave Macmillan; 2012.

48. Yee N, Bailenson JN, Ducheneaut N. The Proteus effect: Implications of transformed digital self-representation on online and offline behavior. Communication Research. 2009; 36:285–312.
49. Goffman E. Interaction ritual: Essays on face-to-face behavior. Garden City, NY: Doubleday/Anchor; 1967. 5–45 p.
50. boyd dm Ellison NB. Social network sites: Definition, history, and scholarship. Journal of Computer-Mediated Communication. 2008; 13:210–30.
51. Bayer JB, Triệu P, Ellison NB. Social media elements, ecologies, and effects. Annual Review of Psychology. 2020; 71:471–97. https://doi.org/10.1146/annurev-psych-010419-050944 PMID: 31518525
52. Lane J. The Digital Street. New York: Oxford University Press; 2018.
53. Earl J, Kimport K. Digitally enabled social change: Activism in the internet age. Cambridge, MA: The MIT Press; 2011.
54. Gershon I. The breakup 2.0: Disconnecting over new media. Ithaca, NY: Cornell University Press; 2010.
55. Skierkowski D, Wood RM. To text or not to text? The importance of text messaging among college-aged youth. Computers in Human Behavior. 2012; 28:744–56.
56. Grinter RE, Eldridge MA. y do tngrs luv 2 txt msg? In: Prinz W, Jarke M, Rogers Y, Schmidt K, Wulf V, editors. ECSCW 2001: Proceedings of the seventh European conference on computer supported cooperative work. Dordrecht: Springer; 2001. p. 219–38.
57. Faulkner X, Culwin F. When fingers do the talking: A study of text messaging. Interacting with Computers. 2005; 17:167–85.
58. McSweeney MA. The pragmatics of text messaging: Making meaning in messages. New York: Routledge; 2018.
59. Drouin M, Landgraff C. Texting, sexting, and attachment in college students' romantic relationships. Computers in Human Behavior. 2012; 28:444–9.
60. Leung L. Unwillingness-to-communicate and college students' motives in SMS mobile messaging. Telematics and Informatics. 2007; 24:115–29.
61. Lin AMY, Tong AHM. Text-messaging cultures of college girls in Hong Kong: SMS as resources for achieving intimacy and gift-exchange with multiple functions. Continuum: Journal of Media & Cultural Studies. 2007; 21:303–15.
62. Ling R, Baron NS. Text messaging and IM: Linguistic comparison of American college data. Journal of Language and Social Psychology. 2007; 26:291–8.
63. Holtgraves T, Paul K. Texting versus talking: An exploration in telecommunication language. Telematics and Informatics. 2013; 30:289–95.
64. Baron NS, Ling R. Necessary smileys & useless periods: Redefining punctuation in electronically-mediated communication. Visible Language. 2011; 45:45–67.
65. Riordan MA. Emojis as tools for emotion work: Communicating affect in text messages. Journal of Language and Social Psychology. 2017; 36:549–67.
66. Carrier LM, Benitez SY. The effect of bilingualism on communication efficiency in text messages (SMS). Multilingua. 2010; 29:167–83.
67. Deumert A, Masinyana SO. Mobile language choices—the use of English and isiXhosa in text messages (SMS): Evidence from a bilingual South African sample. English World-Wide. 2008; 29:117–47.
68. Dietz S, Henrich C. Texting as a distraction to learning in college students. Computers in Human Behavior. 2014; 36:163–7.
69. Murdock KK. Texting while stressed: Implications for students' burnout, sleep, and well-being. Psychology of Popular Media Culture. 2013; 2:207–21.
70. Kingsbury M, Coplan RJ. RU mad @ me? Social anxiety and interpretation of ambiguous text messages. Computers in Human Behavior. 2016; 54:368–79.
71. Turkle S. Alone together: Why we expect more from technology and less from each other. New York: Basic Books; 2011.
72. Crystal D. Txtng: The gr8 db8. Oxford: Oxford University Press; 2008.
73. Johnson AJ, Haigh MM, Becker JAH, Craig EA, Wigley S. College students' use of relational management strategies in email in long-distance and geographically close relationships. Journal of Computer-Mediated Communication. 2008; 13:381–404.
74. Stafford L, Kline SL, Dimmick J. Home e-mail: Relational maintenance and gratification opportunities. Journal of Broadcasting & Electronic Media. 1999; 43:659–69.

75. Wilding R. 'Virtual' intimacies? Families communicating across transnational contexts. Global Networks. 2006; 6:125–42.

76. Skovholt K, Grønning A, Kankaanranta A. The communicative functions of emoticons in workplace e-mails:: -). Journal of Computer-Mediated Communication. 2014; 19:780–97.

77. Baron NS. Letters by phone or speech by other means: The linguistics of email. Language & Communication. 1998; 18:133–70.

78. McCormick NB, McCormick JW. Computer friends and foes: Content of undergraduates' electronic mail. Computers in Human Behavior. 1992; 8:379–405.

79. Garton L, Wellman B. Social impacts of electronic mail in organizations: A review of the research literature. Annals of the International Communication Association. 1995; 18:434–53.

80. Baym NK, Zhang YB, Lin M-C. Social interactions across media: Interpersonal communication on the internet, telephone and face-to-face. New Media & Society. 2004; 6:299–318.

81. Wells TM, Dennis AR. To email or not to email: The impact of media on psychophysiological responses and emotional content in utilitarian and romantic communication. Computers in Human Behavior. 2016; 54:1–9.

82. Colley A, Todd Z. Gender-linked differences in the style and content of e-mails to friends. Journal of Language and Social Psychology. 2002; 21:380–92.

83. Debrand CC, Johnson JJ. Gender differences in email and instant messaging: A study of undergraduate business information systems students. Journal of Computer Information Systems. 2008; 48:20–30.

84. Marmaros D, Sacerdote B. How do friendships form? Quarterly Journal of Economics. 2006; 121:79–119.

85. Sevtsuk A, Chancey B, Basu R, Mazzarello M. Spatial structure of workplace and communication between colleagues: A study of E-mail exchange and spatial relatedness on the MIT campus. Social Networks. 2022; 70:295–305.

86. Menchik DA, Tian X. Putting social context into text: The semiotics of e-mail interaction. American Journal of Sociology. 2008; 114:332–70.

87. Srivastava SB, Goldberg A, Manian VG, Potts C. Enculturation trajectories: Language, cultural adaptation, and individual outcomes in organizations. Management Science. 2018; 64:1348–64.

88. Riordan MA, Kreuz RJ. Cues in computer-mediated communication: A corpus analysis. Computers in Human Behavior. 2010; 26:1806–17.

89. Tong ST, Walther JB. Relational maintenance and CMC. In: Wright KB, Webb LM, editors. Computer-mediated communication in personal relationships. New York: Peter Lang Publishing; 2011. p. 98–118.

90. Quan-Haase A, Wellman B. Hyperconnected net work. In: Heckscher C, Adler P, editors. The Firm as a Collaborative Community: Reconstructing Trust in the Knowledge Economy. New York: Oxford University Pres; 2006.

91. Dainton M, Aylor B. Patterns of communication channel use in the maintenance of long-distance relationships. Communication Research Reports. 2002; 19:118–29.

92. Rabby MK. Relational maintenance and the influence of commitment in online and offline relationships. Communication Studies. 2007; 58:315–37.

93. Wellman B, Wortley S. Different strokes from different folks: Community ties and social support. American Journal of Sociology. 1990; 96:558–88.

94. Baym NK. Personal connections in the digital age. Cambridge, UK: Polity; 2010.

95. Burt RS. Brokerage and closure: An introduction to social capital. New York: Oxford University Press; 2005.

96. Mok D, Wellman B, Carrasco J. Does distance matter in the age of the internet? Urban Studies. 2010; 47:2747–83.

97. Kim H, Kim GJ, Park HW, Rice RE. Configurations of relationships in different media: Ftf, email, instant messenger, mobile phone, and SMS. Journal of Computer-Mediated Communication. 2007; 12:1183–207.

98. Thornberg R, Charmaz K. Grounded theory and theoretical coding. In: Flick U, editor. The SAGE handbook of qualitative data analysis. London: SAGE; 2014. p. 153–69.

99. Corbin J, Strauss A. Basics of qualitative research: Techniques and procedures for developing grounded theory. 4th edition ed. Los Angeles: SAGE; 2015.

100. Charmaz K. Constructing grounded theory. 2nd edition ed. Los Angeles: SAGE; 2014.

101. Hancock JT, Landrigan C, Silver C, editors. Expressing emotion in text-based communication. Proceedings of the ACM Conference on Human Factors in Computing Systems (CHI 2007); 2007.

102. Bearman P, Parigi P. Cloning headless frogs and other important matters: Conversation topics and network structure. Social Forces. 2004; 83:535–57.

103. Dresner E, Herring SC. Functions of the nonverbal in CMC: Emoticons and illocutionary force. Communication Theory. 2010; 20:249–68.

104. Carey J. Paralanguage in computer mediated communication. In: Sondheimer NK, editor. The 18th annual meeting of the association for computational linguistics and parasession on topics in interactive discourse: Proceedings of the conference. Philadelphia: University of Pennsylvania; 1980. p. 67–9.

105. Crystal D. Language and the internet. Cambridge: Cambridge University Press; 2001.

106. Darics E. Non-verbal signalling in digital discourse: The case of letter repetition. Discourse, Context & Media. 2013; 2:141–8.

107. Darics E. Politeness in computer-mediated discourse of a virtual team. Journal of Politeness Research. 2010; 6:129–50.

108. Walther JB, D'Addario KP. The impacts of emoticons on message interpretation in computer-mediated communication. Social Science Computer Review. 2001; 19:324–47.

109. Garrison A, Remley D, Thomas P, Wierszewski E. Conventional faces: Emoticons in instant messaging discourse. Computers and Composition. 2011; 28:112–25.

110. Derks D, Bos AER, von Grumbkow J. Emoticons and online message interpretation. Social Science Computer Review. 2008; 26:379–88.

111. Lo S-K. The nonverbal communication functions of emoticons in computer-mediated communication. CyberPsychology & Behavior. 2008; 11:595–7. https://doi.org/10.1089/cpb.2007.0132 PMID: 18817486

112. Riordan MA, Kreuz RJ. Emotion encoding and interpretation in computer-mediated communication: Reasons for use. Computers in Human Behavior. 2010; 26:1667–73.

113. Gibson DR. Taking turns and talking ties: Networks and conversational interaction. American Journal of Sociology. 2005; 110:1561–97.

114. Hesse BW, Werner CM, Altman I. Temporal aspects of computer-mediated communication. Computers in Human Behavior. 1988; 4:147–65.

115. Davis JL, Jurgenson N. Context collapse: Theorizing context collusions and collisions. Information, Communication & Society. 2014; 17:476–85.

116. boyd d. Social network sites as networked publics: Affordances, dynamics, and implications. In: Papacharissi Z, editor. A networked self: Identity, community, and culture on social network sites. New York: Routledge; 2011. p. 39–58.

117. Baym NK, boyd d. Socially mediated publicness: An introduction. Journal of Broadcasting & Electronic Media. 2012; 56:320–9.

118. Sillence E, Baber C. Integrated digital communities: Combining web-based interaction with text messaging to develop a system for encouraging group communication and competition. Interacting with Computers. 2004; 16:93–113.

119. Borgatti SP, Everett MG, Freeman LC. Ucinet 6 for Windows: Software for Social Network Analysis. Harvard, MA: Analytic Technologies; 2002.

120. Stafford L. Maintaining long-distance and cross-residential relationships. New York: Routledge; 2005.

121. Simmel G. On Individuality and Social Forms. Chicago: University of Chicago Press; 1971.

122. Ito M. Mobile phones, Japanese youth, and the re-placement of social contact. In: Ling R, Pedersen PE, editors. Mobile Communications: Re-negotiation of the Social Sphere. London: Springer; 2005. p. 131–48.

123. Ling R, Yttri B. Hyper-coordination via mobile phones in Norway. In: Katz JE, Aakhus M, editors. Perpetual contact: Mobile communication, private talk, public performance. Cambridge: Cambridge University Press; 2002. p. 139–69.

124. Durkheim E. The Division of Labor in Society. New York: The Free Press; [1893] 1984.

125. Wellman B, Hampton K. Living networked on and offline. Contemporary Sociology. 1999; 28:648–54.

126. Joinson AN. Self-disclosure in computer-mediated communication: The role of self-awareness and visual anonymity. European Journal of Social Psychology. 2001; 31:177–92.

127. Bargh JA, McKenna KYA, Fitzsimons GM. Can you see the real me? Activation and expression of the 'true self' on the internet. Journal of Social Issues. 2002; 58:33–48.

128. Dunbar RIM, Arnaboldi V, Conti M, Passarella A. The structure of online social networks mirrors those in the offline world. Social Networks. 2015; 43:39–47.

129. Turkle S. Reclaiming Conversation: The Power of Talk in a Digital Age. New York: Penguin Books; 2016.

130. Perrin A, Kumar M. About three-in-ten U.S. adults say they are 'almost constantly' online. Washington, DC: Pew Research Center, 2019.

131. Glaser BG. Doing grounded theory: Issues and discussions. Mill Valley, CA: Sociology Press; 1998.

132. Evans JA, Aceves P. Machine translation: Mining text for social theory. Annual Review of Sociology. 2016; 42:21–50.

133. Lazer D, Radford J. Data ex machina: Introduction to big data. Annual Review of Sociology. 2017; 43:19–39.

134. Rogers EM. Progress, problems and prospects for network research: Investigating relationships in the age of electronic communication technologies. Social Networks. 1987; 9:285–310.
